# Aging-Induced Changes in Optical Behavior and Surface Morphology of Additively and Subtractively Manufactured Dental Materials

**DOI:** 10.3390/dj14040210

**Published:** 2026-04-03

**Authors:** Georgiana Osiceanu, Roxana Diana Vasiliu, Flavia Roxana Bejan, Mihaela Ionela Gherban, Liliana Porojan

**Affiliations:** 1 Department of Dental Prostheses Technology (Dental Technology), Center for Advanced Technologies in Dental Prosthodontics, Doctoral School Faculty of Dental Medicine, “Victor Babes” University of Medicine and Pharmacy Timisoara, Eftimie Murgu Sq. No. 2, 300041 Timisoara, Romania; 2 Department of Dental Prostheses Technology (Dental Technology), Center for Advanced Technologies in Dental Prosthodontics, Faculty of Dental Medicine, “Victor Babes” University of Medicine and Pharmacy Timisoara, Eftimie Murgu Sq. No. 2, 300041 Timisoara, Romania; roxana.vasiliu@umft.ro (R.D.V.); flavia.toma@umft.ro (F.R.B.); sliliana@umft.ro (L.P.); 3 National Institute for Research and Development in Electrochemistry and Condensed Matter, 300569 Timisoara, Romania; mihaelabirdeanu@gmail.com

**Keywords:** CAD-CAM, subtractive technology, additive technology, surface treatment, surface roughness, glaze, mechanical polishing, in vitro degradation

## Abstract

**Background/Objectives**: Modern dentistry focuses on the ongoing development of digital alternative technologies and the study of the properties of these innovative materials is deemed essential. Therefore, the objectives of this study were to evaluate the optical and surface characteristics of six computer-aided design/Computer-Aided Manufacturing (CAD-CAM) dental materials, both subtractive and additive, in relation to in vitro degradation. **Methods**: CAD-CAM dental materials, subtractively processed (Vita Enamic, Cerasmart, Brilliant and Tetric) and additively manufactured (Saremco Crowntec and Voco C&B), were prepared to standard dimensions of 14 × 10 × 1 mm, with baseline measurements taken prior to, and after, the degradation procedures, consisting of immersion in an ADA-recommended staining broth, artificial aging (thermocycling), and the combined effects of staining and in vitro aging. Additionally, two different surface treatments were investigated (polished and glazed). **Results**: The poorest color stability was observed for Tetric glazed specimens (mean value 25.585) subjected to staining, while the best performance was recorded for Brilliant polished Control (average value of 0.781). The staining procedure produced the most pronounced color changes. Surface treatment did not significantly affect color stability, and surface roughness was not influenced by either the degradation method or the surface treatment (*p* > 0.05). Atomic Force Microscopy (AFM) evaluation revealed superior performance of the glazed surfaces, characterized by lower nanoroughness values compared with polished surfaces and a smoother surface appearance. **Conclusions:** The staining potential of staining broth was demonstrated in this study, with the highest values recorded after the staining procedures. In addition, the influence of artificial aging alone and artificial aging combined with staining was investigated, providing relevant results for a better clinical approach. Moreover, surface treatment demonstrated reliability and therefore clinical applicability.

## 1. Introduction

From a historical perspective, computer-aided design and manufacturing (known as CAD-CAM) technology dates back to the 1970s, with its first use in dentistry appearing in the 1990s [1]. In this branch of medicine, the use of CAD-CAM has grown in response to ongoing technological progress, in line with advancements in other fields in contemporary times [2]. Additionally, there has been an increased demand for less time-consuming, cost-effective solutions that offer color stability and better surface preservation for dental materials. CAD-CAM systems offer several benefits, such as the ability to provide fast, precise, and cost-free personalized indirect restorations [3]. The dental materials that can be used with CAD-CAM technology to produce restoration include composite resins and ceramics, such as lithium disilicate, feldspathic, and leucite-reinforced glass ceramics [4].

Therefore, the recent development of CAD-CAM technology is fully justified, with attention focused on the two categories of CAD-CAM dental materials: subtractive and, more recently introduced, additive. The new 3D technology allows the production of challenging dental anatomy that would otherwise be difficult to create, using less material—since the dental restoration is built from the beginning rather than milled from a block, reducing waste—and lowering costs [5]. By utilizing a wide range of 3D printers and diverse printing materials, various dental fields can be addressed: prosthodontics (temporary crowns, full dentures, and partial fixed prostheses), surgery (surgical guides), and occlusology (occlusal splints) [6].

Among subtractive classification materials, Vita Enamic, Brilliant Crios, Tetric CAD, and Cerasmart are representative of this category. The recent advancements of this technology resulted in the appearance of Vita Enamic, classified as a PICN material (polymer-infiltrated ceramic network) and representing the first hybrid dental ceramic [7,8].

The most common monomer incorporated into CAD/CAM composite blocks is urethane dimethacrylate (UDMA), which has been shown to have reduced water sorption and solubility compared with Bisphenol A-glycidyl methacrylate (Bis-GMA) [9]. According to previous studies, Bis-GMA resins demonstrate higher degrees of water sorption in comparison with UDMA, whereas Ethoxylated Bisphenol A Dimethacrylate (a Bis-EMA resin, which does not contain the hydroxyl groups found in Bis-GMA nor the urethane linkages found in UDMA), showed the most reduced water absorption values [10].

Introducing different types of fillers—such as ceramic particles, silica glass, and pre-polymerized components—has been shown to improve the properties of dental composites [11].

Regarding the 3D innovation field, there are many 3D technologies, and Stereolithography (SLA) and Digital Light Processing (DLP) are two of them. A powerful Light-Emitting Diode (LED) is utilized to polymerize a large area at once, in two directions (x/y axis), in DLP technology [12,13]. Because of this, DLP proves to be faster than SLA [12,13]. Also, an important advantage of both technologies is that they can use many types of resin [12]. However, studies reported that 3D-printed technology performed poorer than traditional materials and subtractive technology in terms of susceptibility to staining, probably due to their disadvantages, such as moisture uptake and slow polymerization [14,15,16,17]. 3D technologies may exceed subtractive methods, as their advantages are well known, including more efficient material use, reduced waste, and improved manufacturing capacity [18].

Regarding the composition of the 3D materials, the main components consist of photoinitiators (3–5 wt%), epoxy and acrylate monomers, which improve the polymerization process, and UV absorbers [19]. The filler amount ranges between 40 nm and 40 μm, the form may be round or irregular, and, in some materials, they can reach up to 60% in terms of volume [20].

The ongoing development of additive technology allows the manufacturing of definitive and short- or long-lasting temporary restorations [21].

Color stability is an important feature of a successful restoration; nowadays, indirect dental restorations should meet patients’ expectations regarding the maintenance of esthetic properties over time. Coloring may be caused by external factors (such as the effects brought by the polymerization process, contact with ultraviolet (UV radiance), humidity, temperature, and dietary colorants ingested daily) and intrinsic factors [22,23].

Among the most common staining liquids reported in numerous investigations are coffee, red wine, and tea [24].

The properties of the internal components of the material summarize the intrinsic factors that determine the amount of external pigmentation, also taking surface smoothness into account [22].

Another crucial parameter in the prevalence of color change and the prevention of bacterial plaque retention is surface roughness [1]. From a clinical point of view, the accepted value of Ra is 0.2 µm [25,26].

In an attempt to overcome these insufficiencies, polishing and glazing are two tested methods. Glaze materials (light-curing in the case of a resin matrix structure) are intended to behave like surface sealants, thus resulting in improved surface characteristics (lower pigmentation, better mechanical properties, regular surface structure, and the absence of defects) [1,27]. They seal flaws and gaps, reducing microleakage and decreasing the polymer’s ability to absorb water [5,28].

There is limited knowledge in the literature regarding the efficiency of surface treatments (polishing vs. glazing), and even fewer studies involve the use of a staining cocktail aimed to reproduce overall exposure to most of the colorants encountered in everyday life and to better simulate the in vivo conditions of the oral environment, including substances such as mucin. Moreover, the literature lacks studies that simultaneously compare both subtractive and additive materials, due to the novelty of these materials. Their comparative performance has not been sufficiently investigated, nor have the ADA recommendations for staining protocols been completely addressed. These outcomes are important, as they can help guide clinicians in selecting the most appropriate approach for different clinical situations. Also, evaluating both surface and optical characteristics provides an overall view of the performance of these materials under the investigated conditions.

Therefore, the objectives of the present study were to test the following null hypotheses: (H0)—There were no significant differences between the polished and glazed surfaces; (H1)—There were no significant differences between the control, stained, and thermocycled groups; and (H2)—There were no significant differences between the materials.

## 2. Materials and Methods

### 2.1. Materials

In this study, we used CAD-CAM subtractively processed dental materials—Vita Enamic (VITA Zahnfabrik, Bad Säckingen, Germany), Cerasmart (GC Corportion, Tokyo, Japan), Tetric (Ivoclar Vivadent, Schaan, Liechtenstein), and Brilliant Crios (Coltene/Whaledent, Alstatten, Switzerland)—and additively processed materials—Saremco Crowntec (Saremco Dental AG, Rebstein, Switzerland) and Voco V-Print C&B Temp (VOCO GmbH, Cuxhaven, Germany). The CAD-CAM blocks used were shade A2 HT and size 14. The materials’ composition and the manufacturers’ information can be identified in Table 1.

#### 2.1.1. Preparation of Subtractive Material Specimens

The blocks were fixed and sectioned using a dental iso-parallelograph device to obtain parallelepiped plates. These specimens were then processed to final dimensions of 14 × 10 × 1 mm to ensure dimensional standardization across all materials, resulting in 120 samples and 240 surfaces. Then, the specimens were polished using abrasive and finished with Fegupol Compo + (Feguramed GmbH, Heppenheim, Germany) paste. The size of the specimens was constantly verified using a digital caliper. All polishing procedures were performed by a single operator to ensure consistency and standardization.

#### 2.1.2. Preparation of Additive Material Specimens

Rectangular plate specimens with uniform dimensions (approximately 14 × 10 × 1 mm) were digitally designed, with a 50 µm layer thickness, and subsequently printed using an Asiga Max 3D printer (Asiga Max UV, Asiga, Sydney, Australia)), resulting in 60 specimens and 120 surfaces. The specimens were then cleaned in an ultrasonic bath using 99% isopropyl alcohol for 3 min, allowed to dry, and photopolymerized in a BB Midi Plus (MECCATRONICORE, Pergine Valsugana, Italy) curing unit for 10 min.

#### 2.1.3. Samples Grouping and Surface Treatment

The 180 samples (360 examined surfaces) were divided into two groups according to surface treatment: finishing with abrasive papers (240–2000 grit) followed by polishing with Compo + paste, and surface conditioning with glaze application. Consequently, the first group underwent conventional finishing and polishing (*n* = 180), while the samples in the second group (*n* = 180) were sandblasted for 10 s with 50 µm Al_2_O_3_ particles (2.5 bar pressure), keeping a distance of 10 mm from the specimen surface, using a sandblasting device Sirio Dental (Sirio Dental S.r.l., Meldola, Forlì-Cesena, Italy). After sandblasting, the specimens were washed in distilled water for 5 min and then air-dried. Following this, G-Multi Primer (GC) was applied to the sandblasted surfaces for 30 s and air-dried. The final step comprised the glazing procedure: Vita Akzent LC was applied with a soft brush in one direction and photopolymerized for 180 s in a polymerization unit, an Asiga Flash Curing Unit (Asiga, Sydney, Australia), according to the manufacturer’s instructions.

#### 2.1.4. Aging Procedures

Regarding the aging procedures, the samples from each material were divided into four groups: Group 1—Control group (specimens were immersed in distilled water for 12 days), Group 2—Staining broth immersion (samples were immersed in staining broth for 12 days), Group 3—Thermocycling (10.000 cycles), and Group 4—Thermocycling + staining broth immersion (10.000 cycles + staining broth immersion for 12 days).

##### Staining Protocol

For the staining procedure, an innovative staining broth based on ADA recommendations [29], reported by a few researchers [30], and adapted for this study was used and consisted of 50 g of soluble coffee (Amigo, Strauss Coffee B.V. Netherlands/Romania market), 266.5 mL of red wine (Domeniile Viticole Tohani, Feteasca Neagra, semi-dry), 25 g of black tea (Fares Bio Vital SRL, Orăștie, Romania), 1 g mucin, 0.75 mL red dye, 0.75 mL yellow dye, 0.0625 g methylparaben, 0.0375 g propylparaben and distilled water, resulting in a total volume of 500 mL staining solution. The staining solution was prepared based on commonly used protocols to simulate clinical conditions. Sixty samples were distributed in glass containers (*n* = 10 per container) with 30 mL of staining broth each and maintained in an incubator at 37 °C for 12 days, with periodic refreshment of the solution to prevent contamination. Color and surface parameters were recorded at baseline and after 12 days of immersion.

##### Thermocycling

Sixty samples were thermocycled for 10.000 cycles in alternating baths between 5 °C and 55 °C (30 s dwell time; 10 s transfer time), to replicate one year of clinical conditions [31]. Color and surface properties were evaluated before and after the thermocycling procedure.

### 2.2. Color Evaluation

The evaluation of color was done using a spectrophotometer (Vita Easy Shade IV, Zahnfabrik, Bad Säckingen, Germany) in CIEL*a*b* system, using D65 standardized lighting, which simulates natural daylight, along with the ultraviolet component [2]. Before the measurement of the color parameters, the device was calibrated to ensure its accuracy.

A WhiBal G7 gray card (White Balance Pocket Card) was used to register values on gray, white and black backgrounds. All three color parameters—L*, a*, and b*—were taken into account when evaluating color changes, with the following interpretations: the L* coordinate (indicating lightness) ranges from 0 (complete black) to 100 (complete white), while a* represents the position on the red–green axis (positive values = red and negative values = green) and b* represents the position on the yellow–blue axis (positive values = yellow and negative values = blue).

Measurements were taken three times for each specimen, and the mean value was calculated.

ΔE*ab was calculated using the following formula:

$$\Delta E_{ab}\;=\;\sqrt{{\left(\Delta L\right)}^{2}+{\left(\Delta a\right)}^{2}+{\left(\Delta b\right)}^{2}}$$

For clinical interpretation, ΔE values were also related to commonly accepted perceptibility thresholds: 0 < ΔE < 1—no color difference is perceived by the observer; 1 < ΔE < 2—the difference can be detected only by a trained observer; 2 < ΔE < 3.5—the difference becomes noticeable even for an untrained observer; 3.5 < ΔE < 5—a clear color difference is perceived; and ΔE > 5—the observer perceives two distinct colors [32].

### 2.3. Surface Roughness Evaluation

Surface texture was assessed with a contact profilometer with a 2 μm stylus, Surftest SJ-201 (Mitutoyo, Kawasaki, Tokyo, Japan), assessing two surface parameters: Ra (μm) (arithmetic mean roughness) and Rz (μm) (maximum vertical surface roughness) values. The procedure used a cut-off value set at 0.8 mm, applying a force of 0.7 N.

### 2.4. Atomic Force Microscopy (AFM)

Surface topography analysis was performed using an atomic force microscope (Nanosurf Easy Scan 2 Advanced Research; Nanosurf, Liestal, Switzerland), allowing the determination of nanoroughness parameters Sa and Sq. Surface topography was analyzed in non-contact mode with a scan area of 2.2 × 2.2 μm.

### 2.5. Statistical Analysis

A power analysis using G*Power software v3.0.10 I (f = 0.40, α = 0.05, and power 80%) indicated that 10 specimens per stage per material are sufficient. Each specimen had two surfaces measured (polished and glazed), resulting in a total of 180 specimens and 360 surfaces, allowing reliable comparisons between stages and surface treatments.

The JASP program (version 0.95.4) was used to perform statistical analysis. With the help of this statistical program, the Shapiro–Wilk test was used to determine the normality of the data, and Levene’s test was used to determine the homogeneity of variances. The Welch ANOVA test was used to identify statistically significant differences, and the Games–Howell post hoc test was employed for multiple comparisons. Pearson correlation analysis was used to correlate changes in color with modifications in surface roughness.

## 3. Results

### 3.1. Color Changes

Regarding the color determination for all categories of materials, under both surface treatments (polished vs. glazed) and across the three stages (control, stained, thermocycled), the mean values and standard deviations can be observed in Table 2. Normality was assessed using the Shapiro–Wilk test.

**Figure 1:**
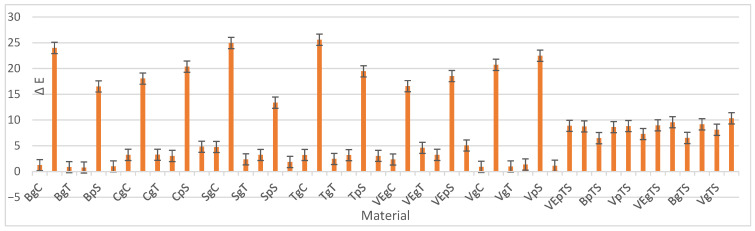
ΔE means and SDs for all materials.

Regarding the mean values and standard deviations of each material, the highest value was recorded for TgS (Tetric glazed Stained), while the lowest was observed for BpC (Brilliant polished Control) (Table 2, Figure 1). Therefore, the material that demonstrated the best performance was Brilliant (Table 2, Figure 1).

Regarding the comparison between manufacturing methods (subtractive vs. additive), a *p*-value of 0.773 was recorded, indicating no statistically significant differences between them. However, the interaction between ΔE, the material, and manufacturing method was statistically significant (*p* < 0.001). Further, different interactions between factors such as the stages, surface treatments, and materials were examined.

As data were normally distributed (Shapiro–Wilk *p* > 0.05 in the majority of cases), but they did not present equality of variances (Levene *p* < 0.001), the values were compared using Welch ANOVA, followed by the Games–Howell Post hoc test. The Welch ANOVA test showed statistically significant differences between the materials regarding the ΔE values (*p* < 0.001). Games–Howell post hoc comparisons revealed statistically significant differences between almost all pairwise combinations. (Supplementary Table S1).

**Figure 2:**
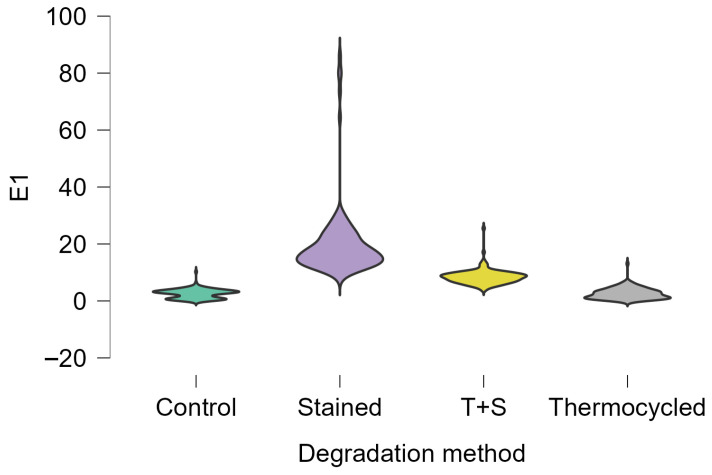
Means and SDs for the four stages (control, stained, thermocycled, and T + S—thermocycled + stained); recorded ΔE1 values of color change after 12 days of staining broth immersion.

In terms of the degradation method, the four stages were compared (control, staining, thermocycling, and staining + thermocycling), with the highest value being recorded for the stained samples (with a recorded mean value 20.049), while the lowest was observed for the control group (mean value of 2.546), followed by the thermocycled group (mean value of 2.620) (Figure 2). This demonstrated the fact that the staining procedure had the greatest impact on the color modifications.

The Welch ANOVA test showed that, for the factors taken into consideration (ΔE values and degradation methods), there were statistically significant differences between the four stages (control, stained, thermocycled, and thermocycled + stained) (*p* < 0.001). Post hoc Games–Howell multiple comparisons between the stages revealed statistically significant differences between all combinations, with the exception of the control and thermocycled groups (Table 3).

For the comparison of the surface treatments (glazed vs. polished), in terms of means and standard deviations, the glazed surfaces registered a higher mean value (8.868 vs. 7.973), but there were no statistically significant differences between the surfaces (*p* = 0.318).

### 3.2. Surface Roughness Assessment

**Figure 3:**
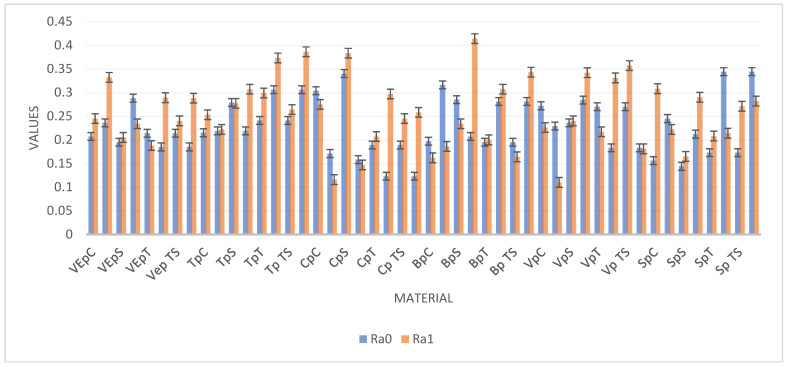
Means and SDs of Ra0 and Ra1 expressed in µm.

The means and standard deviations for the surface roughness parameters, Ra0 and Ra1, for all materials, stages, and surface treatments can be observed in Figure 3. The highest surface roughness value was registered by BgS (Brilliant glazed stained, 0.414 µm) and the lowest by VgC (Voco glazed Control, 0.11 µm).

A paired Student’s *t*-test was applied to evaluate statistically significant differences between Ra0 and Ra1, with a *p* value of 0.003.

As Levene’s test indicated inequality of variances (*p*-0.001), Welch’s ANOVA was applied, showing that there were not statistically significant differences between groups of materials, degradation methods or surface treatments (*p* > 0.05).

A Pearson correlation analysis revealed a very weak positive correlation between surface roughness (Ra) and color change (ΔE) (r = 0.073, *p*-0.113).

### 3.3. Atomic Force Microscopy (AFM) Evaluation

Polished and glazed samples from all the three stages (control, stained, and thermocycled + stained) were scanned in six different areas to obtain three-dimensional surface images. For the polished surfaces, AFM analysis revealed the presence of numerous surface defects, including voids and protuberances (Table 4). In contrast, the glazed surfaces exhibited relatively smoother topography compared with the polished ones, likely due to the protective surface characteristics of the applied glaze (Table 5). Nevertheless, operator-related defects were also observed in the glazed specimens, such as bubble incorporation, highlighting manipulation-related issues induced by the operator (clinician).

In terms of nanoroughness, the Sa parameter (average nanoroughness value) was recorded. The Shapiro–Wilk test indicated a normal distribution of the data (*p* > 0.05); however, Levene’s test revealed unequal variances (*p* < 0.05). Therefore, Welch’s ANOVA was applied to assess statistically significant differences. The means and standard deviations are presented in Table 6, with VEpC (Vita Enamic polished control) showing the highest mean value (30.200 nm) and BgS (Brilliant glazed stained) the lowest (mean value: 1.480 nm). The Games–Howell post hoc test revealed significant differences between almost all pairwise combinations of materials.

**Figure 4:**
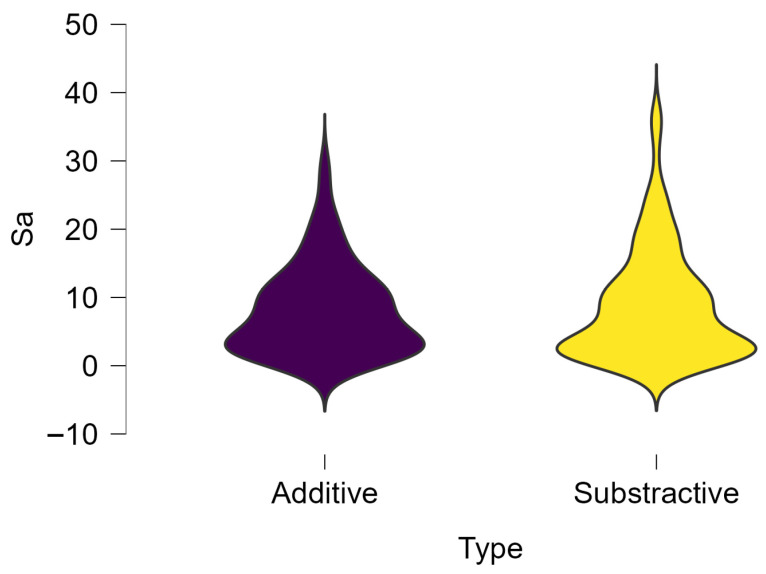
Descriptive Statistics regarding additive vs. subtractive types; Sa—arithmetical mean surface roughness.

Regarding the type of material (subtractive vs. additive), the data were non-normally distributed (*p* < 0.001) but showed homogeneity of variances (*p* > 0.05). Higher values were recorded for the subtractive category (Figure 4); however, no statistically significant differences were observed between the two manufacturing types, as indicated by the Mann–Whitney U test (*p* = 0.829). There were no statistically significant differences between the degradation methods (control, stained, thermocycled, and thermocycled + stained, *p* = 0.112).

**Figure 5:**
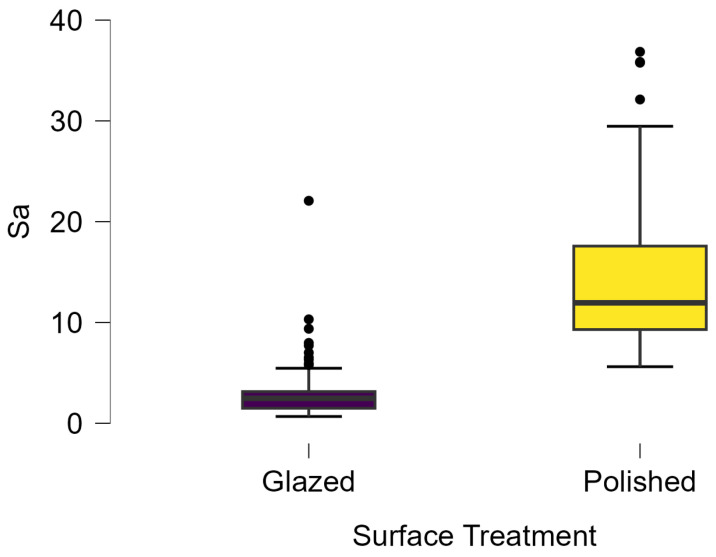
Descriptive statistics regarding surface treatments; Sa—arithmetical mean surface roughness.

Regarding surface treatment, the means and standard deviations are presented in Figure 5. Polished specimens presented substantially higher values (mean Sa = 14.026), compared with the glazed ones (mean Sa = 2.985). A statistically significant difference was observed between surface treatments (glazed vs. polished; *p* < 0.001).

Furthermore, the means and standard deviations for material and stage are presented in Table 7.

## 4. Discussion

One popular method to mimic the in vitro effects of aging on dental restorations is thermocycling [3]. Thermocycling mimics temperature changes in the oral cavity, creating stress in the material because the resin and filler expand and contract at different rates [33]. According to previous studies, thermocycling may alter the material’s color and surface texture [34]. This aging method is associated with physical degradation, such as fatigue, wear and chemical degradation, including hydrolytic, enzymatic, and temperature-related changes affecting the internal composition of the material [35]. It has been stated that the optical properties of resin composites are modified by the thermocycling process in combination with immersion in a coloring medium [36]. In this study, thermocycling combined with the staining procedure resulted in much higher discoloration values than thermocycling alone, supporting the conclusion presented in the previously mentioned study. The second null hypothesis was rejected, as significant differences were observed between the stages. The 10,000 cycles were selected based on the statement that 10,000 cycles simulate one year of clinical service [31].

In the present study, a staining broth related to ADA recommendations was selected due to its high staining potential, taking into consideration that it includes the most common coloring liquids [29]. Few adapted studies were found in the literature, as this staining method has not yet been sufficiently studied at the current state of knowledge [30,37,38,39]. Coffee and tea, especially black tea, commonly used as coloring agents, are well known for their strong staining potential [40]. The tannins in tea help the staining molecules stick more easily to the surface of restorations [40]. Pigments (melanin), organic acids (chlorogenic acid), and polyphenols (tannins) are components present in coffee [41]. Natural chromogens, which have large molecular sizes and low polarity, can bind to the polymer network of resin composites, leading to discoloration [42,43]. Red wine is also known for staining dental restorations, mainly because of compounds like tannins and anthocyanins [44]. However, it has been shown that common drinks like red wine or tea, because of their acidity, can cause changes in color, which may either exaggerate or underestimate how much discoloration occurs when they are used alone [45,46]. Therefore, by combining the staining effects of each added component, the staining solution used in this research is more reliable; all the ingredients help replicate the conditions of human beverage intake and the addition of mucin helps the pellicle to develop [38]. Also, distilled water was chosen as the control, as previous investigations have demonstrated that it does not produce significant color change, with any color alteration being attributed to a phenomenon known as internal coloring, resulting from the absorption of water molecules by the resin composites [47,48].

Based on the assumption that one day of laboratory staining simulates one month of in vivo conditions, an immersion period of 12 days was considered to represent one year in the oral cavity [49,50].

In this study, the CIE L*a*b* system was used to determine color modifications using a spectrophotometer, as it is a standard method for color quantification in dental medicine [44]. The selected background for determining the color parameters was gray, as it is known to have a reduced influence on lightness perception [51]. For clinical interpretation, anterior research has indicated that ΔE values below 1.5 are unnoticeable to the human eye, whereas values exceeding 3.3 are considered clinically unacceptable [52]. In this study, almost all values exceeded the clinically acceptable threshold and the level detectable by the human eye, with the exception of BpC, VpC, VpT, BgC, VgC, BgT, VgT, VEpTS, TpTS, BpTS, VegTS, TgTS, BgTS, CgTS, VgTS, and SgTS.

Also, taking into account the perceptibility thresholds, most values were situated in the ranges of “two different colors” and “a clear color difference perceived”.

Another important characteristic is surface roughness, which may lead to bacterial plaque retention, color change, and the formation and spreading of fractures within the restoration [53,54]. So, achieving a smooth surface through polishing procedures or glazing application is crucial [55,56]. One study comparing the surface roughness of six resin composites after artificial aging found that Tetric CAD was the roughest, likely due to its larger filler particle size surface [57]. The interconnected ceramic network and reduced filler particle size of Vita Enamic are thought to contribute to its superior surface performance [58]. In this study, no statistically significant differences were observed between the materials, regardless of surface treatment or aging procedures, leading to the conclusion that the aging procedures and surface treatments did not affect surface roughness. Therefore, the first null hypothesis was accepted.

Regarding the AFM evaluation, the superiority of the glazed surfaces was demonstrated. Additionally, the nanoroughness assessment revealed voids within the external structure of the materials and surface defects, some of which could be attributed to clinician manipulation. In conclusion, in terms of nanoroughness, the first null hypothesis was rejected.

The hydrophobicity and hydrophilicity of monomers, along with their water uptake, play an important role in color changes [59]. Bis-GMA is known to be more hydrophilic, while UDMA, TEGMA and Bis-EMA are less susceptible to water sorption [60].

One study investigated the relationship between different monomers and their susceptibility to absorb water [61]. They reported that TEGDMA showed the highest tendency, followed by Bis-GMA, Bis-EMA, and UDMA, which showed the weakest connection [61]. Hydrophilic resin materials tend to absorb water as well as pigments from foods and beverages [44]. In one study evaluating CAD-CAM blocks in staining solutions, Brilliant Crios showed the greatest degree of color modification, which was probably associated with its monomers, Bis-GMA and TEGDMA [47]. In the current research, the high rates of discoloration of Tetric can be attributed to the monomers within the internal structure of the resin. There were statistically significant differences between the materials; therefore, the third null hypothesis was also rejected.

Moreover, filler particle characteristics—including size, type, and content—play a significant role in the resistance to discoloration of composite resins [47,62]. Particles with smaller dimensions are less susceptible to water sorption and consequently to lower surface roughness [63]. According to one study, the better performance of Vita Enamic in terms of discoloration was attributed to its lower Bis-GMA composition and higher filler content [64,65]. Moreover, the degree of color change is strongly correlated with monomer type, as reported in the literature [65]. In the present research, the overall lower values observed for Vita Enamic could be attributed to its high filler proportion.

In one study comparing Cerasmart and Vita Enamic, Vita Enamic demonstrated better performance than Cerasmart in terms of color change [66]. In this study, the values representing the staining degradation method for each material showed pronounced color changes compared with the control, thermocycling, and combined thermocycling and staining procedures.

The limitations of this study include the potential influence of glaze thickness on the optical outcomes, which was not considered; the in vitro study design; the absence of mechanical parameter evaluations; and the limited number of specimens included in the AFM analysis.

Additionally, the importance of optical stability and its relationship with mechanical performance has been evaluated in several previous studies, highlighting the need to correlate mechanical properties with the long-term performance of both additive and subtractive materials [67]. The correlation between these parameters not being studied in the current work represents another limitation.

Future perspectives consist of the following:

1. Investigating other aging methods and other categories of materials.
2. Considering other correlations, for example, correlations with mechanical properties.
3. SEM evaluation of the material microstructure before and after aging procedures.

## 5. Conclusions

Within the limitations of this study, the following can be concluded:

1. Regarding the materials’ performance, the worst performance in terms of maintaining color stability was recorded by a glazed subtractive material, during the staining stage, whereas the best performance was observed for a glazed subtractive material in the thermocycling stage. No significant differences were recorded between the manufacturing methods, additive vs. subtractive.
2. Regarding the most pronounced color changes across the degradation stages, the staining degradation method produced the most notable changes (ΔE mean = 20.049). Statistically significant differences were observed between the stages (*p* < 0.001 except for the control–thermocycled comparison).
3. Regarding the surface treatment, glazed surfaces showed poorer performance than polished surfaces; however, no statistically significant differences were found between the two treatments.
4. Regarding the AFM evaluation, better performance of the glazed surfaces was revealed.
5. Surface roughness was not affected by the degradation method and was not influenced by the different surface treatments.

## Figures and Tables

**Table 1:** CAD CAM materials used in this study.

Material	Type	Manufacturer	Composition	Shade/Translucency
Vita Enamic (VE)	Hybrid Ceramic	VITA Zahnfabrik, Bad Säckingen, Germany	Feldspar ceramic, enriched with aluminum oxide 86%, UDMA, TEGMA	A2/MT
Cerasmart (C)	CAD/CAM composite resin	GC Corportion, Tokyo, Japan	Silica, barium glass 71%, UDMA,3 Bis-MEPP, DMA	A2/MT
Tetric CAD (T)	Composite resin	Ivoclar Vivadent, Liechtenstein	Nano-filled 70% with barium glass and silicon dioxide, Bis-GMA, Bis-EMA, TEGDMA, UDMA	A2
Brilliant Crios (B)	Composite resin	Coltene/Whaledent, Alstatten, Switzerland	Amorphous silica particles (<20 nm) and glassy ceramic, barium particles (<1.0 µm), BisGMA, UDMA, TEGDMA	A2
Saremco Crowntec (S)	Composite based resin	Saremco Dental AG Rebstein, Switzerland ethoxylated and 2-methylprop-2enoic acid, silanized dental glass, Pyrogenic silica, initiators	Esterification products of 4,4′-isopropylidiphenol. Total content of inorganic fillers (particle size 0.7 μm) is 30–50% by mass	A2
Voco V-Print C&B Temp(V)		VOCO GmbH, Cuxhaven. Germany	Ceramic-filled (26 wt% inorganic fillers) hybrid. Material with aliphatic urethane dimethacrylate (>10–25%), aliphatic acrylate (>2.5–10%), triethylene glycol dimethacrylate (>2.5–10%), diphenyl (2,4,6-trimethylbenzoyl)phosphine oxide (max. 2.5%)	A2
Vita Akzent	VITA Zahnfabrik, Methyl methacrylate and multifunctional methacrylates	Bad Säckingen, Germany	Wt% 30–40, urethane (meth-)acrylates 40–60, silicon dioxide 8–11, Ethyl-phenyl (2,4,6-trimethylbenzoyl)phosphinate 2–6 Other < 1 Pigments < 2 5	

**Table 2:** Descriptive statistics—means, SDs of ΔE1, and *p*-values of the Shapiro–Wilk test for normality assumption.

		Valid	Missing	Mean	Std. Deviation	Shapiro–Wilk	*p*-Value of Shapiro–Wilk	Minimum	Maximum
ΔE1	VEpC	10	0	3.242	0.422	0.866	0.089	2.786	3.920
ΔE1	TpC	10	0	3.188	0.796	0.944	0.604	1.594	4.409
ΔE1	BpC	10	0	0.781	0.292	0.941	0.568	0.436	1.345
ΔE1	CpC	10	0	3.023	1.186	0.851	0.060	0.141	4.585
ΔE1	VpC	10	0	1.372	1.742	0.536	<0.001	0.447	6.230
ΔE1	SpC	11	0	3.236	0.589	0.900	0.184	2.371	4.509
ΔE1	VEpS	10	0	18.527	19.424	0.465	<0.001	8.389	73.52
ΔE1	TpS	10	0	19.460	15.888	0.444	<0.001	11.52	64.51
ΔE1	BpS	10	0	16.527	4.774	0.933	0.478	9.557	24.38
ΔE1	CpS	10	0	20.356	21.395	0.458	<0.001	10.61	80.95
ΔE1	VpS	10	0	22.477	22.884	0.510	<0.001	11.98	86.03
ΔE1	SpS	10	0	13.385	2.431	0.911	0.291	10.50	18.75
ΔE1	VEpT	10	0	5.069	0.741	0.955	0.729	3.964	6.143
ΔE1	TpT	10	0	3.047	0.667	0.983	0.980	1.863	4.259
ΔE1	BpT	10	0	0.993	0.620	0.871	0.102	0.412	2.343
ΔE1	CpT	10	0	4.818	3.083	0.595	<0.001	2.927	13.10
ΔE1	VpT	10	0	1.123	0.458	0.926	0.414	0.608	2.062
ΔE1	SpT	10	0	1.857	1.234	0.808	0.018	0.640	4.142
ΔE1	VEgC	10	0	2.339	1.354	0.805	0.017	0.424	4.057
ΔE1	TgC	10	0	3.218	1.180	0.853	0.063	0.374	4.843
ΔE1	BgC	10	0	1.243	1.234	0.761	0.005	0.283	3.736
ΔE1	CgC	10	0	3.236	0.995	0.863	0.083	1.049	4.168
ΔE1	VgC	10	0	0.904	0.660	0.859	0.075	0.245	2.047
ΔE1	SgC	10	0	4.780	2.059	0.706	0.001	3.046	10.23
ΔE1	VEgS	10	0	16.572	2.568	0.966	0.856	12.23	21.73
ΔE1	TgS	10	0	25.585	19.160	0.554	<0.001	13.53	79.05
ΔE1	BgS	10	0	23.992	5.906	0.922	0.377	12.46	30.95
ΔE1	CgS	10	0	18.048	2.769	0.949	0.656	12.88	23.21
ΔE1	VgS	10	0	20.713	1.871	0.905	0.250	18.11	23.41
ΔE1	SgS	10	0	24.952	2.960	0.967	0.866	20.59	30.27
ΔE1	VEgT	10	0	4.606	1.276	0.909	0.272	1.926	6.675
ΔE1	TgT	10	0	2.439	0.506	0.939	0.537	1.828	3.305
ΔE1	BgT	10	0	0.863	0.526	0.944	0.599	0.245	1.879
ΔE1	CgT	10	0	3.267	1.254	0.798	0.014	1.612	6.426
ΔE1	VgT	10	0	0.980	1.024	0.697	<0.001	0.141	3.713
ΔE1	SgT	10	0	2.371	1.538	0.927	0.422	0.469	4.833
ΔE1	VEpTS	10	0	8.874	1.172	0.938	0.529	6.979	10.44
ΔE1	TpTS	10	0	8.761	0.473	0.962	0.808	8.002	9.455
ΔE1	BpTS	10	0	6.485	1.049	0.924	0.396	5.049	7.896
ΔE1	CpTS	10	0	8.624	2.033	0.906	0.257	6.326	12.58
ΔE1	VpTS	10	0	8.829	6.478	0.679	<0.001	3.994	25.51
ΔE1	SpTS	10	0	7.292	3.642	0.680	<0.001	3.986	17.11
ΔE1	VEgTS	10	0	8.983	0.974	0.962	0.806	7.153	10.33
ΔE1	TgTS	10	0	9.583	0.600	0.926	0.412	8.571	10.29
ΔE1	BgTS	10	0	6.528	0.956	0.972	0.910	5.205	8.301
ΔE1	CgTS	10	0	9.181	1.301	0.939	0.543	6.751	11.50
ΔE1	VgTS	10	0	8.112	1.709	0.853	0.063	6.364	12.14
ΔE1	SgTS	10	0	10.339	1.702	0.911	0.287	8.165	13.22

VE—Vita Enamic, T—Tetric, C—Cerasmart, B—Brilliant V—Voco, S—Saremco, p—polished, g—glazed, C—control, S—stained, T—thermocycled, TS—stained+ thermocycled

**Table 3:** Games–Howell post hoc comparison and degradation method.

Comparison	Mean Difference	SE	t	df	p_tukey_
Control−Stained	−17.503	1.197	−14.627	122.7	<0.001
Control−Thermocycled	−0.073	0.230	−0.319	230.6	0.989
Control−Thermocycled + Stained	−5.920	0.278	−21.260	199.3	<0.001
Stained–Thermocycled	17.430	1.201	14.518	124.3	<0.001
Stained–Thermocycled + Stained	11.583	1.211	9.567	128.4	<0.001
Thermocycled–Thermocycled + Stained	−5.847	0.295	−19.821	220.3	<0.001

**Table 4:** The AFM images of the polished surfaces for all materials.

	Control Polished	Stained Polished	Thermocycled + Stained Polished
VITA ENAMIC	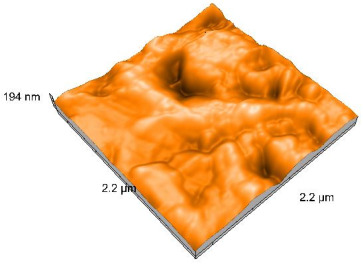	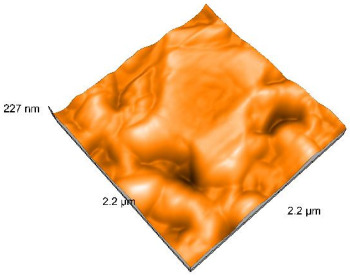	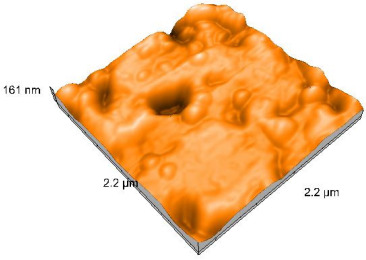
TETRIC	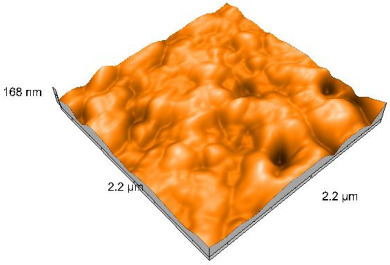	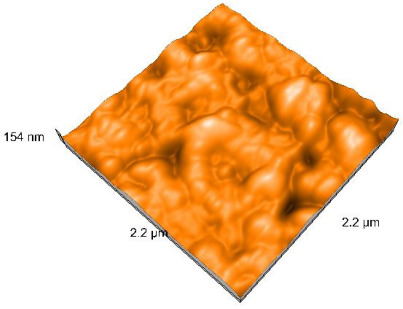	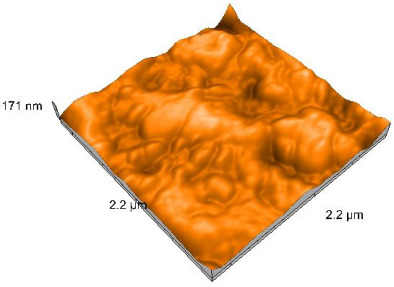
CERASMART	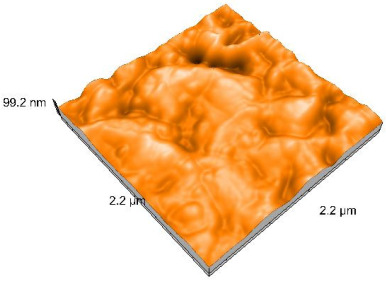	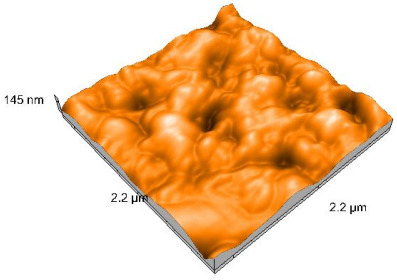	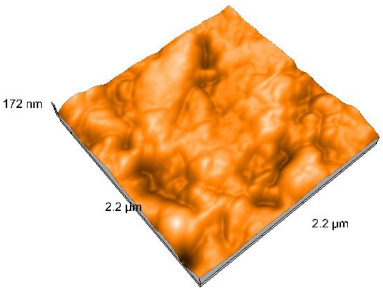
BRILLIANT	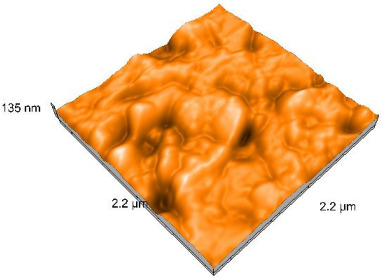	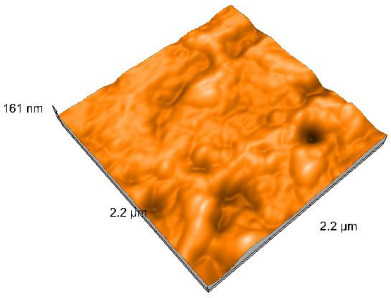	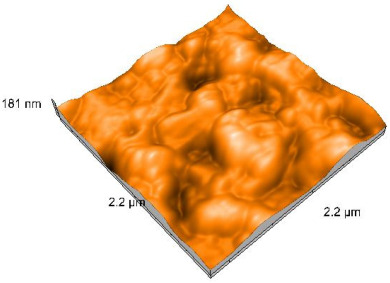
VOCO	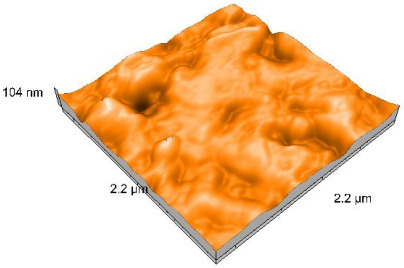	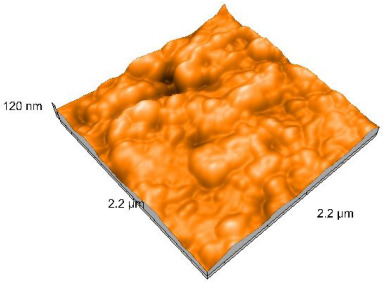	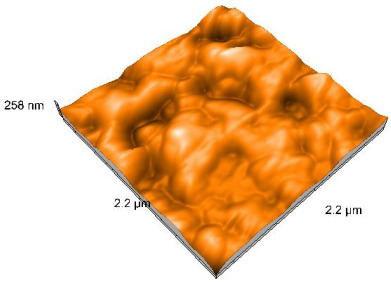
SAREMCO	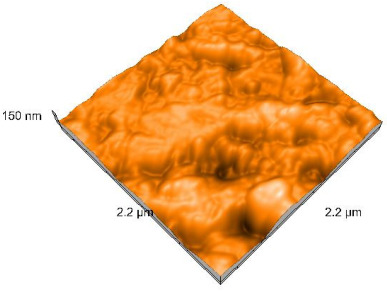	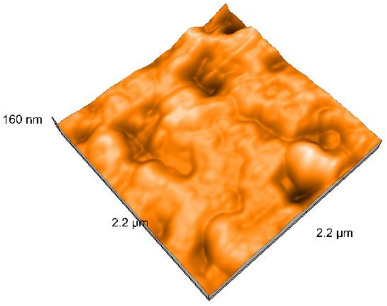	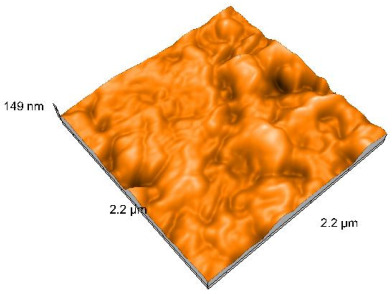

**Table 5:** The AFM images of the glazed surfaces of all materials.

	Control Glazed	Stained Glazed	Thermocycled + Stained Glazed
VITA ENAMIC	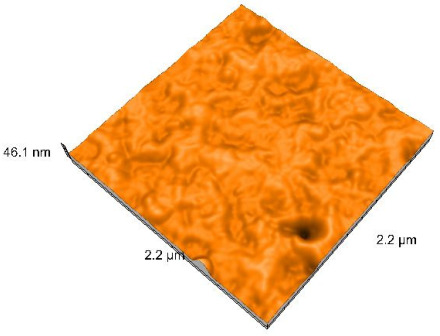	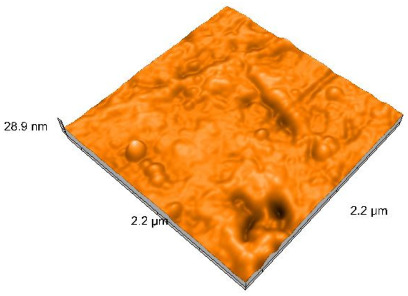	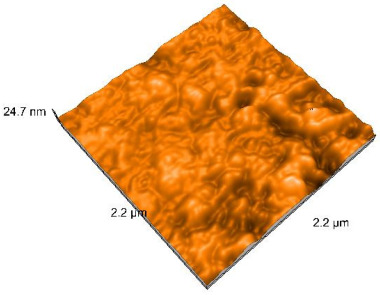
TETRIC	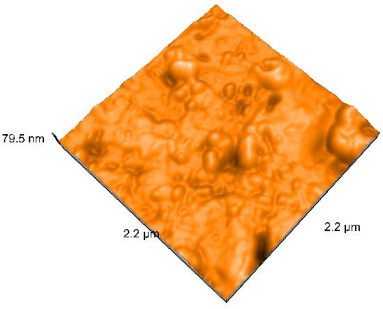	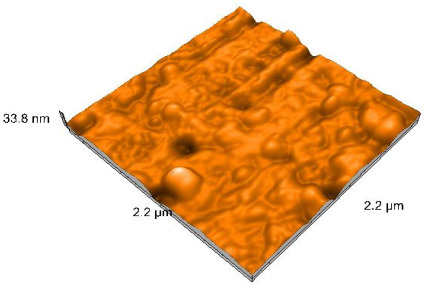	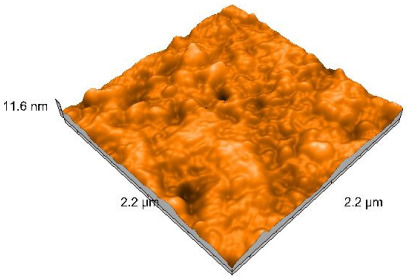
CERASMART	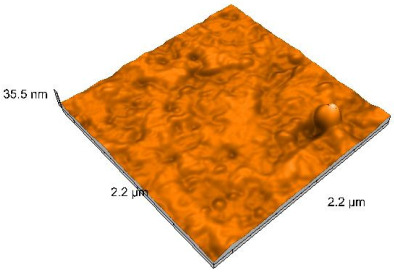	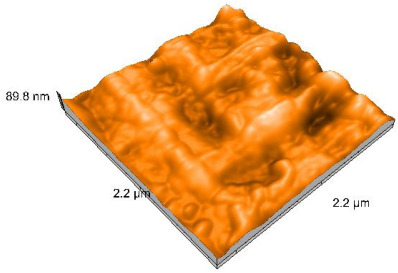	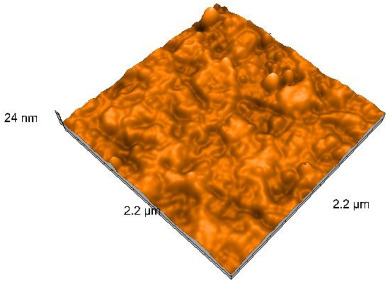
BRILLIANT	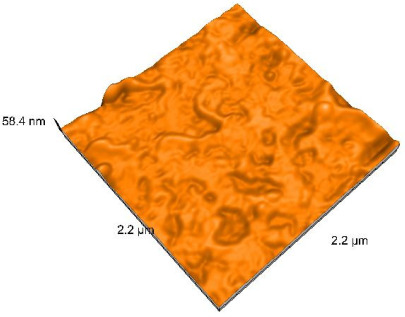	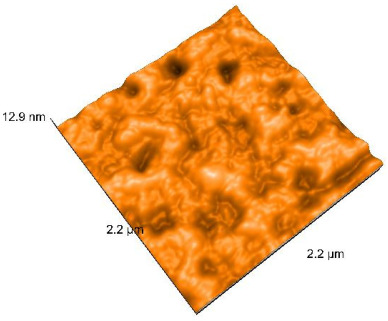	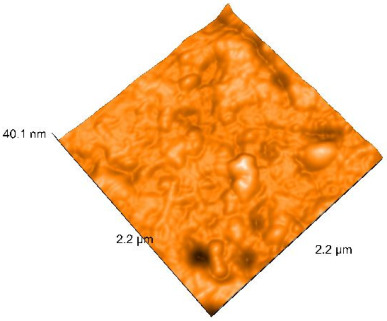
VOCO	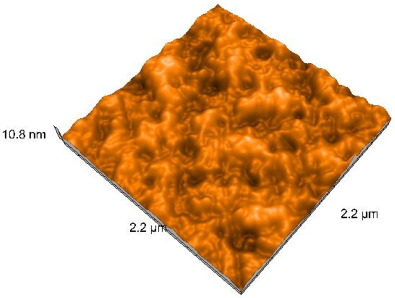	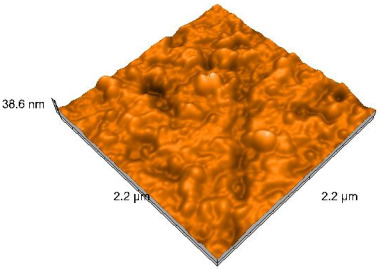	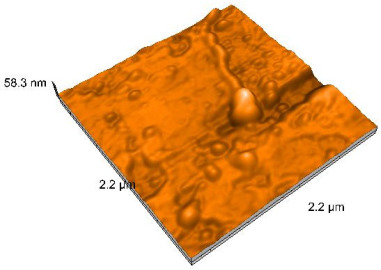
SAREMCO	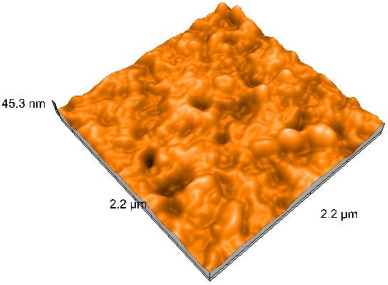	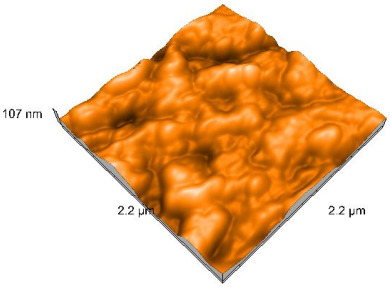	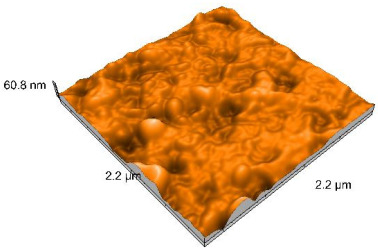

**Table 6:** Descriptive statistics—means, SDs of Sa, and *p*-values of the Shapiro–Wilk test for normality assumption.

		Valid	Missing	Mean	Std. Deviation	Shapiro–Wilk	*p*-Value of Shapiro–Wilk	Minimum	Maximum
Sa	TpC	6	0	20.246	4.085	0.854	0.168	16.36	27.91
Sa	TgC	6	0	5.906	1.492	0.947	0.719	4.054	7.971
Sa	TpS	6	0	9.678	0.976	0.933	0.601	8.307	11.31
Sa	TgS	6	0	2.341	0.904	0.915	0.472	1.466	3.788
Sa	TpTS	6	0	20.475	2.931	0.936	0.628	16.69	24.23
Sa	TgTS	6	0	1.593	0.68	0.853	0.167	1.009	2.663
Sa	VpC	6	0	11.231	1.831	0.812	0.074	9.253	14.71
Sa	VgC	6	0	4.707	8.525	0.55	<0.001	0.701	22.07
Sa	VpS	6	0	6.711	1.075	0.909	0.432	5.625	8.496
Sa	VgS	6	0	2.648	0.443	0.911	0.444	2.036	3.13
Sa	VpTS	6	0	13.46	2.767	0.956	0.788	10.13	17.94
Sa	VgTS	6	0	1.845	0.808	0.878	0.258	0.962	2.952
Sa	VEpC	6	0	30.2	6.593	0.786	0.043	23.15	36.86
Sa	VEgC	6	0	1.967	0.582	0.982	0.962	1.109	2.843
Sa	VEpS	6	0	15.093	2.896	0.932	0.593	12	19.67
Sa	VEgS	6	0	2.312	0.588	0.966	0.866	1.467	3.058
Sa	VEpTS	6	0	11.031	2.204	0.951	0.745	7.622	13.62
Sa	VEgTS	6	0	2.229	0.743	0.922	0.52	0.913	3.119
Sa	BpC	6	0	19.959	7.151	0.922	0.52	12.61	32.12
Sa	BgC	6	0	2.869	1.45	0.93	0.582	1.359	5.18
Sa	BpS	6	0	9.782	3.278	0.978	0.942	5.612	14.55
Sa	BgS	6	0	1.48	0.851	0.856	0.175	0.671	2.663
Sa	BpTS	6	0	8.897	2.085	0.953	0.768	6.281	11.95
Sa	BgTS	6	0	2.327	0.927	0.942	0.679	0.792	3.413
Sa	CpC	6	0	12.364	3.652	0.923	0.529	7.792	18.75
Sa	CgC	6	0	2.419	0.678	0.741	0.016	1.089	2.938
Sa	CpS	6	0	11.034	2.569	0.855	0.172	8.394	15.85
Sa	CgS	6	0	4.369	1.596	0.846	0.147	1.383	5.779
Sa	CpTS	6	0	8.708	1.517	0.873	0.238	7.157	10.75
Sa	CgTS	6	0	1.493	0.473	0.883	0.285	1.039	2.156
Sa	SpC	6	0	18.13	5.799	0.955	0.784	10.68	26.02
Sa	SgC	6	0	2.178	0.773	0.894	0.342	1.009	2.906
Sa	SpS	6	0	8.126	1.756	0.948	0.721	5.865	10.52
Sa	SgS	6	0	4.714	1.17	0.881	0.274	3.577	6.317
Sa	SpTS	6	0	17.345	6.28	0.78	0.039	12.23	29.47
Sa	SgTS	6	0	6.33	3.273	0.915	0.467	2.514	10.31

**Table 7:** Descriptive statistics—means and standard deviations (SDs) of Sa according to surface treatment and material.

Surface Treatment + Material	N	Mean	SD	SE	Coefficient of Variation
Polished TpC	6	20.246	4.085	1.668	0.202
Glazed TgC	6	5.906	1.492	0.609	0.253
Polished TpS	6	9.678	0.976	0.398	0.101
Glazed TgS	6	2.341	0.904	0.369	0.386
Polished TpTS	6	20.475	2.931	1.197	0.143
Glazed TgTS	6	1.593	0.68	0.278	0.427
Polished VpC	6	11.231	1.831	0.747	0.163
Glazed VgC	6	4.707	8.525	3.48	1.811
Polished VpS	6	6.711	1.075	0.439	0.16
Glazed VgS	6	2.648	0.443	0.181	0.167
Polished VpTS	6	13.46	2.767	1.13	0.206
Glazed VgTS	6	1.845	0.808	0.33	0.438
Polished VEpC	6	30.2	6.593	2.692	0.218
Glazed VEgC	6	1.967	0.582	0.237	0.296
Polished VEpS	6	15.093	2.896	1.182	0.192
Glazed VEgS	6	2.312	0.588	0.24	0.254
Polished VEpTS	6	11.031	2.204	0.9	0.2
Glazed VEgTS	6	2.229	0.743	0.303	0.333
Polished BpC	6	19.959	7.151	2.92	0.358
Glazed BgC	6	2.869	1.45	0.592	0.505
Polished BpS	6	9.782	3.278	1.338	0.335
Glazed BgS	6	1.48	0.851	0.347	0.575
Polished BpTS	6	8.897	2.085	0.851	0.234
Glazed BgTS	6	2.327	0.927	0.378	0.398
Polished CpC	6	12.364	3.652	1.491	0.295
Glazed CgC	6	2.419	0.678	0.277	0.28
Polished CpS	6	11.034	2.569	1.049	0.233
Glazed CgS	6	4.369	1.596	0.652	0.365
Polished CpTS	6	8.708	1.517	0.619	0.174
Glazed CgTS	6	1.493	0.473	0.193	0.317
Polished SpC	6	18.13	5.799	2.367	0.32
Glazed SgC	6	2.178	0.773	0.315	0.355
Polished SpS	6	8.126	1.756	0.717	0.216
Glazed SgS	6	4.714	1.17	0.478	0.248
Polished SpTS	6	17.345	6.28	2.564	0.362
Glazed SgTS	6	6.33	3.273	1.336	0.517

## Data Availability

The original contributions presented in this study are included in the article and Supplementary Materials. Further inquiries can be directed to the corresponding author.

## Supplementary Materials

The following supporting information can be downloaded at: https://www.mdpi.com/article/10.3390/dj14040210/s1, Table S1: Games-Howell Post Hoc Comparisons regarding the Material.
