# Supplementary material for: Aging-Induced Changes in Optical Behavior and Surface Morphology of Additively and Subtractively Manufactured Dental Materials
Source: Dent J (Basel). 2026 Apr 3;14(4):210. doi: 10.3390/dj14040210 (PMC13115512; doi:10.3390/dj14040210)
Supplement: Supplementary file 1 [file dentistry-14-00210-s001.zip › dentistry-4176639-supplementary.pdf]

Article

Aging-Induced Changes in Surface Morphology and Optical Behavior of Additively and Subtractively Manufactured Dental Materials

Georgiana Osiceanu [1,*], Flavia Roxana Bejan[2], Roxana Diana Vasiliu[2] Mihaela Ionela Gherban[3] and Liliana Porojan [2]

[1] Department of Dental Prostheses Technology (Dental Technology), Center for Advanced Technologies in Dental Prosthodontics, Doctoral School Faculty of Dental Medicine, "Victor Babes" University of Medicine and Pharmacy Timisoara, Eftimie Murgu Sq. No. 2, 300041 Timisoara, Romania; georgiana.osiceanu@umft.ro (G.O)

[2] Department of Dental Prostheses Technology (Dental Technology), Center for Advanced Technologies in Dental Prosthodontics, Faculty of Dental Medicine, "Victor Babes" University of Medicine and Pharmacy Timisoara, Eftimie Murgu Sq. No. 2, 300041 Timisoara, Romania; sliliana@umft.ro (L.P.)

[3]National Institute for Research and Development in Electrochemistry and Condensed Matter, 300569 Timisoara, Romania; mihaelabirdeanu@gmail.com

* Correspondence: georgiana.osiceanu@umft.ro

Table S1 Games-Howell Post Hoc Comparisons regarding the Material

Comparison	Mean Difference	SE	t	df	p_{tukey}
BgC – BgS	-22.749	1.908	-11.922	9.784	< .001
BgC – BgT	0.380	0.424	0.895	12.165	1.000
BgC – BpC	0.462	0.401	1.153	10.006	1.000
BgC – BpS	-15.284	1.559	-9.802	10.197	< .001
BgC - BpT	0.250	0.437	0.572	13.267	1.000
BgC - CgC	-1.993	0.501	-3.976	17.225	.175
BgC - CgS	-16.805	0.959	-17.530	12.438	< .001
BgC - CgT	-2.024	0.556	-3.639	17.995	.284
BgC - CpC	-1.780	0.541	-3.289	17.972	.450
BgC - CpS	-19.113	6.777	-2.820	9.060	.701
BgC - CpT	-3.575	1.050	-3.405	11.811	.420

Comparison	Mean Difference	SE	t	df	p_{tukey}
BgC - SgC	-3.537	0.759	-4.661	14.726	.068
BgC - SgS	-23.709	1.014	-23.382	12.036	< .001
BgC - SgT	-1.128	0.623	-1.810	17.193	.996
BgC - SpC	-1.988	0.437	-4.551	13.281	.091
BgC - SpS	-12.142	0.862	-14.084	13.348	< .001
BgC - SpT	-0.614	0.552	-1.112	18.000	1.000
BgC - TgC	-1.975	0.540	-3.658	17.964	.276
BgC - TgS	-24.342	6.071	-4.009	9.075	.241
BgC - TgT	-1.196	0.422	-2.837	11.941	.697
BgC - TpC	-1.945	0.464	-4.188	15.389	.136
BgC - TpS	-18.217	5.039	-3.615	9.109	.360
BgC - TpT	-1.804	0.444	-4.067	13.845	.175
BgC - VEgC	-1.096	0.579	-1.892	17.847	.993
BgC - VEgS	-15.329	0.901	-17.013	12.944	< .001
BgC - VEgT	-3.363	0.561	-5.991	17.979	.005
BgC - VEpC	-1.999	0.412	-4.849	11.080	.075
BgC - VEpS	-17.284	6.155	-2.808	9.073	.706
BgC - VEpT	-3.826	0.455	-8.407	14.746	< .001
BgC - VgC	0.339	0.442	0.766	13.761	1.000
BgC - VgS	-19.470	0.709	-27.469	15.582	< .001
BgC - VgT	0.263	0.507	0.518	17.407	1.000
BgC - VpC	-0.129	0.675	-0.191	16.213	1.000
BgC - VpS	-21.234	7.247	-2.930	9.052	.650
BgC - VpT	0.120	0.416	0.288	11.431	1.000
BgC - VEpTS	-7.631	0.538	-14.178	17.953	< .001

Comparison	Mean Difference	SE	t	df	p_{tukey}
BgC - TpTS	-7.518	0.418	-17.993	11.589	< .001
BgC - BpTS	-5.242	0.512	-10.234	17.548	< .001
BgC - CpTS	-7.381	0.752	-9.816	14.839	< .001
BgC - VpTS	-7.586	2.085	-3.638	9.652	.346
BgC - SpTS	-6.049	1.216	-4.975	11.039	.064
BgC - VEgTS	-7.740	0.497	-15.570	17.082	< .001
BgC - TgTS	-8.340	0.434	-19.226	13.027	< .001
BgC - BgTS	-5.285	0.494	-10.709	16.941	< .001
BgC - CgTS	-7.938	0.567	-13.997	17.949	< .001
BgC - VgTS	-6.869	0.667	-10.306	16.378	< .001
BgC - SgTS	-9.096	0.665	-13.685	16.414	< .001
BgS - BgT	23.128	1.875	12.334	9.143	< .001
BgS - BpC	23.211	1.870	12.412	9.044	< .001
BgS - BpS	7.465	2.402	3.108	17.242	.551
BgS - BpT	22.998	1.878	12.246	9.198	< .001
BgS - CgC	20.756	1.894	10.958	9.510	< .001
BgS - CgS	5.943	2.063	2.881	12.774	.676
BgS - CgT	20.724	1.909	10.854	9.809	< .001
BgS - CpC	20.969	1.905	11.007	9.725	< .001
BgS - CpS	3.635	7.019	0.518	10.364	1.000
BgS - CpT	19.174	2.107	9.101	13.565	< .001
BgS - SgC	19.211	1.978	9.713	11.155	< .001
BgS - SgS	-0.961	2.089	-0.460	13.252	1.000
BgS - SgT	21.620	1.930	11.202	10.214	< .001
BgS - SpC	20.761	1.878	11.055	9.199	< .001

Comparison	Mean Difference	SE	t	df	p_{tukey}
BgS - SpS	10.607	2.020	5.252	11.964	.039
BgS - SpT	22.135	1.908	11.601	9.785	< .001
BgS - TgC	20.774	1.905	10.907	9.717	< .001
BgS - TgS	-1.593	6.340	-0.251	10.695	1.000
BgS - TgT	21.552	1.875	11.497	9.132	< .001
BgS - TpC	20.804	1.885	11.039	9.327	< .001
BgS - TpS	4.532	5.360	0.845	11.441	1.000
BgS - TpT	20.945	1.880	11.143	9.229	< .001
BgS - VEgC	21.653	1.916	11.300	9.943	< .001
BgS - VEgS	7.420	2.037	3.643	12.286	.319
BgS - VEgT	19.385	1.911	10.145	9.839	< .001
BgS - VEpC	20.749	1.873	11.081	9.092	< .001
BgS - VEpS	5.464	6.420	0.851	10.650	1.000
BgS - VEpT	18.922	1.882	10.052	9.283	< .001
BgS - VgC	23.088	1.879	12.285	9.225	< .001
BgS - VgS	3.279	1.959	1.674	10.789	.997
BgS - VgT	23.011	1.896	12.139	9.540	< .001
BgS - VpC	22.620	1.947	11.616	10.554	< .001
BgS - VpS	1.515	7.474	0.203	10.194	1.000
BgS - VpT	22.868	1.873	12.207	9.108	< .001
BgS - VEpTS	15.117	1.904	7.939	9.708	.003
BgS - TpTS	15.231	1.874	8.129	9.115	.003
BgS - BpTS	17.507	1.897	9.229	9.568	< .001
BgS - CpTS	15.367	1.975	7.780	11.103	.002
BgS - VpTS	15.162	2.772	5.469	17.848	.012

Comparison	Mean Difference	SE	t	df	p_{tukey}
BgS - SpTS	16.700	2.194	7.611	14.979	< .001
BgS - VEgTS	15.008	1.893	7.928	9.489	.003
BgS - TgTS	14.408	1.877	7.675	9.186	.005
BgS - BgTS	17.463	1.892	9.230	9.471	< .001
BgS - CgTS	14.811	1.913	7.744	9.872	.003
BgS - VgTS	15.879	1.944	8.167	10.496	.002
BgS - SgTS	13.653	1.944	7.024	10.484	.006
BgT - BpC	0.083	0.190	0.435	14.070	1.000
BgT - BpS	-15.663	1.519	-10.314	9.218	< .001
BgT - BpT	-0.130	0.257	-0.506	17.537	1.000
BgT - CgC	-2.373	0.356	-6.668	13.666	.004
BgT - CgS	-17.185	0.891	-19.281	9.648	< .001
BgT - CgT	-2.404	0.430	-5.592	12.072	.024
BgT - CpC	-2.160	0.410	-5.264	12.407	.036
BgT - CpS	-19.493	6.768	-2.880	9.011	.673
BgT - CpT	-3.955	0.989	-3.999	9.523	.237
BgT - SgC	-3.917	0.672	-5.830	10.169	.025
BgT - SgS	-24.089	0.951	-25.341	9.568	< .001
BgT - SgT	-1.508	0.514	-2.935	11.077	.648
BgT - SpC	-2.367	0.257	-9.203	17.527	< .001
BgT - SpS	-12.522	0.787	-15.920	9.840	< .001
BgT - SpT	-0.994	0.424	-2.342	12.163	.907
BgT - TgC	-2.355	0.409	-5.764	12.439	.018
BgT - TgS	-24.722	6.061	-4.079	9.014	.224
BgT - TgT	-1.576	0.231	-6.832	17.973	< .001

Comparison	Mean Difference	SE	t	df	p_{tukey}
BgT - TpC	-2.325	0.302	-7.704	15.596	< .001
BgT - TpS	-18.597	5.027	-3.699	9.020	.332
BgT - TpT	-2.183	0.269	-8.130	17.072	< .001
BgT - VEgC	-1.476	0.459	-3.213	11.656	.509
BgT - VEgS	-15.709	0.829	-18.949	9.753	< .001
BgT - VEgT	-3.743	0.437	-8.574	11.970	< .001
BgT - VEpC	-2.379	0.213	-11.156	17.197	< .001
BgT - VEpS	-17.664	6.145	-2.875	9.013	.676
BgT - VEpT	-4.206	0.287	-14.638	16.231	< .001
BgT - VgC	-0.041	0.267	-0.152	17.145	1.000
BgT - VgS	-19.849	0.615	-32.294	10.413	< .001
BgT - VgT	-0.117	0.364	-0.322	13.441	1.000
BgT - VpC	-0.509	0.575	-0.884	10.626	1.000
BgT - VpS	-21.614	7.238	-2.986	9.010	.624
BgT - VpT	-0.260	0.220	-1.179	17.664	1.000
BgT - VEpTS	-8.011	0.406	-19.714	12.480	< .001
BgT - TpTS	-7.898	0.224	-35.314	17.801	< .001
BgT - BpTS	-5.622	0.371	-15.146	13.252	< .001
BgT - CpTS	-7.761	0.664	-11.689	10.199	< .001
BgT - VpTS	-7.966	2.055	-3.876	9.119	.276
BgT - SpTS	-6.429	1.164	-5.525	9.375	.043
BgT - VEgTS	-8.120	0.350	-23.193	13.833	< .001
BgT - TgTS	-8.720	0.252	-34.574	17.698	< .001
BgT - BgTS	-5.665	0.345	-16.423	13.992	< .001
BgT - CgTS	-8.318	0.444	-18.739	11.862	< .001

Comparison	Mean Difference	SE	t	df	p_{tukey}
BgT - VgTS	-7.249	0.565	-12.821	10.689	< .001
BgT - SgTS	-9.476	0.563	-16.824	10.703	< .001
BpC - BpS	-15.746	1.512	-10.411	9.067	< .001
BpC - BpT	-0.213	0.217	-0.982	12.812	1.000
BpC - CgC	-2.455	0.328	-7.490	10.541	.003
BpC - CgS	-17.268	0.881	-19.611	9.200	< .001
BpC - CgT	-2.487	0.407	-6.109	9.974	.019
BpC - CpC	-2.242	0.386	-5.805	10.088	.027
BpC - CpS	-19.576	6.766	-2.893	9.003	.667
BpC - CpT	-4.037	0.979	-4.123	9.162	.212
BpC - SgC	-4.000	0.658	-6.083	9.362	.023
BpC - SgS	-24.172	0.940	-25.702	9.175	< .001
BpC - SgT	-1.591	0.495	-3.214	9.649	.518
BpC - SpC	-2.450	0.217	-11.295	12.800	< .001
BpC - SpS	-12.604	0.774	-16.279	9.260	< .001
BpC - SpT	-1.076	0.401	-2.684	10.005	.765
BpC - TgC	-2.437	0.384	-6.341	10.099	.014
BpC - TgS	-24.805	6.060	-4.093	9.004	.221
BpC - TgT	-1.659	0.185	-8.983	14.403	< .001
BpC - TpC	-2.407	0.268	-8.976	11.379	< .001
BpC - TpS	-18.679	5.025	-3.717	9.006	.326
BpC - TpT	-2.266	0.230	-9.843	12.330	< .001
BpC - VEgC	-1.558	0.438	-3.558	9.836	.373
BpC - VEgS	-15.791	0.817	-19.319	9.233	< .001
BpC - VEgT	-3.826	0.414	-9.239	9.940	< .001

Comparison	Mean Difference	SE	t	df	p_{tukey}
BpC - VEpC	-2.462	0.162	-15.164	16.008	< .001
BpC - VEpS	-17.747	6.143	-2.889	9.004	.669
BpC - VEpT	-4.289	0.252	-17.027	11.730	< .001
BpC - VgC	-0.123	0.228	-0.540	12.395	1.000
BpC - VgS	-19.932	0.599	-33.282	9.438	< .001
BpC - VgT	-0.200	0.337	-0.593	10.456	1.000
BpC - VpC	-0.591	0.559	-1.059	9.505	1.000
BpC - VpS	-21.696	7.237	-2.998	9.003	.619
BpC - VpT	-0.343	0.172	-1.995	15.287	.983
BpC - VEpTS	-8.094	0.382	-21.182	10.113	< .001
BpC - TpTS	-7.981	0.176	-45.403	14.994	< .001
BpC - BpTS	-5.705	0.344	-16.561	10.386	< .001
BpC - CpTS	-7.844	0.649	-12.078	9.371	< .001
BpC - VpTS	-8.049	2.051	-3.925	9.037	.264
BpC - SpTS	-6.512	1.155	-5.636	9.116	.040
BpC - VEgTS	-8.203	0.322	-25.502	10.605	< .001
BpC - TgTS	-8.803	0.211	-41.734	13.043	< .001
BpC - BgTS	-5.748	0.316	-18.188	10.667	< .001
BpC - CgTS	-8.400	0.422	-19.916	9.904	< .001
BpC - VgTS	-7.332	0.548	-13.373	9.525	< .001
BpC - SgTS	-9.558	0.546	-17.507	9.530	< .001
BpS - BpT	15.533	1.522	10.204	9.303	< .001
BpS - CgC	13.291	1.542	8.619	9.780	.001
BpS - CgS	-1.522	1.745	-0.872	14.441	1.000
BpS - CgT	13.259	1.561	8.495	10.235	.001

Comparison	Mean Difference	SE	t	df	p_{tukey}
BpS - CpC	13.504	1.555	8.681	10.107	.001
BpS - CpS	-3.830	6.932	-0.552	9.894	1.000
BpS - CpT	11.709	1.797	6.516	15.395	.003
BpS - SgC	11.746	1.644	7.145	12.236	.003
BpS - SgS	-8.426	1.776	-4.744	15.028	.058
BpS - SgT	14.155	1.586	8.925	10.848	< .001
BpS - SpC	13.296	1.522	8.734	9.304	.002
BpS - SpS	3.142	1.694	1.855	13.374	.992
BpS - SpT	14.670	1.559	9.408	10.198	< .001
BpS - TgC	13.309	1.555	8.559	10.096	.001
BpS - TgS	-9.059	6.244	-1.451	10.113	1.000
BpS - TgT	14.087	1.518	9.280	9.202	.001
BpS - TpC	13.339	1.530	8.716	9.500	.001
BpS - TpS	-2.933	5.246	-0.559	10.612	1.000
BpS - TpT	13.480	1.524	8.844	9.351	.001
BpS - VEgC	14.188	1.569	9.042	10.438	< .001
BpS - VEgS	-0.045	1.714	-0.026	13.807	1.000
BpS - VEgT	11.920	1.563	7.628	10.280	.003
BpS - VEpC	13.284	1.515	8.766	9.141	.002
BpS - VEpS	-2.001	6.325	-0.316	10.083	1.000
BpS - VEpT	11.457	1.528	7.500	9.433	.005
BpS - VgC	15.623	1.524	10.252	9.344	< .001
BpS - VgS	-4.186	1.621	-2.582	11.702	.816
BpS - VgT	15.546	1.544	10.069	9.826	< .001
BpS - VpC	15.155	1.607	9.431	11.356	< .001

Comparison	Mean Difference	SE	t	df	p_{tukey}
BpS - VpS	-5.950	7.392	-0.805	9.782	1.000
BpS - VpT	15.403	1.517	10.157	9.165	< .001
BpS - VEpTS	7.652	1.554	4.923	10.082	.077
BpS - TpTS	7.765	1.517	5.119	9.177	.070
BpS - BpTS	10.042	1.546	6.497	9.868	.013
BpS - CpTS	7.902	1.641	4.816	12.160	.069
BpS - VpTS	7.697	2.545	3.025	16.548	.599
BpS - SpTS	9.235	1.899	4.864	16.825	.041
BpS - VEgTS	7.543	1.541	4.896	9.749	.083
BpS - TgTS	6.943	1.521	4.564	9.284	.129
BpS - BgTS	9.998	1.540	6.494	9.720	.013
BpS - CgTS	7.346	1.565	4.695	10.331	.099
BpS - VgTS	8.414	1.603	5.248	11.270	.043
BpS - SgTS	6.188	1.603	3.861	11.251	.255
BpT - CgC	-2.243	0.371	-6.052	15.069	.007
BpT - CgS	-17.055	0.897	-19.007	9.899	< .001
BpT - CgT	-2.274	0.442	-5.143	13.149	.039
BpT - CpC	-2.030	0.423	-4.796	13.572	.062
BpT - CpS	-19.363	6.769	-2.861	9.015	.682
BpT - CpT	-3.825	0.994	-3.846	9.726	.277
BpT - SgC	-3.787	0.680	-5.570	10.617	.032
BpT - SgS	-23.959	0.956	-25.056	9.787	< .001
BpT - SgT	-1.378	0.524	-2.629	11.847	.797
BpT - SpC	-2.237	0.277	-8.068	18.000	< .001
BpT - SpS	-12.391	0.793	-15.620	10.164	< .001

Comparison	Mean Difference	SE	t	df	p_{tukey}
BpT - SpT	-0.864	0.437	-1.978	13.265	.982
BpT - TgC	-2.225	0.421	-5.279	13.612	.030
BpT - TgS	-24.592	6.062	-4.057	9.019	.230
BpT - TgT	-1.446	0.253	-5.718	17.306	.008
BpT - TpC	-2.195	0.319	-6.879	16.975	.001
BpT - TpS	-18.467	5.028	-3.673	9.027	.341
BpT - TpT	-2.053	0.288	-7.133	17.903	< .001
BpT - VEgC	-1.345	0.471	-2.858	12.612	.688
BpT - VEgS	-15.579	0.835	-18.647	10.044	< .001
BpT - VEgT	-3.613	0.449	-8.053	13.018	< .001
BpT - VEpC	-2.249	0.237	-9.487	15.877	< .001
BpT - VEpS	-17.534	6.145	-2.853	9.018	.686
BpT - VEpT	-4.076	0.305	-13.345	17.452	< .001
BpT - VgC	0.089	0.286	0.312	17.929	1.000
BpT - VgS	-19.719	0.623	-31.636	10.950	< .001
BpT - VgT	0.013	0.378	0.034	14.814	1.000
BpT - VpC	-0.379	0.585	-0.648	11.240	1.000
BpT - VpS	-21.484	7.239	-2.968	9.013	.633
BpT - VpT	-0.130	0.244	-0.533	16.570	1.000
BpT - VEpTS	-7.881	0.419	-18.793	13.662	< .001
BpT - TpTS	-7.768	0.246	-31.516	16.830	< .001
BpT - BpTS	-5.492	0.385	-14.251	14.594	< .001
BpT - CpTS	-7.631	0.672	-11.356	10.658	< .001
BpT - VpTS	-7.836	2.058	-3.808	9.165	.296
BpT - SpTS	-6.299	1.168	-5.392	9.520	.048

Comparison	Mean Difference	SE	t	df	p_{tukey}
BpT - VEgTS	-7.990	0.365	-21.883	15.255	< .001
BpT - TgTS	-8.590	0.273	-31.505	17.981	< .001
BpT - BgTS	-5.535	0.360	-15.368	15.429	< .001
BpT - CgTS	-8.188	0.456	-17.963	12.880	< .001
BpT - VgTS	-7.119	0.575	-12.384	11.326	< .001
BpT - SgTS	-9.346	0.573	-16.320	11.345	< .001
CgC - CgS	-14.813	0.930	-15.920	11.285	< .001
CgC - CgT	-0.031	0.506	-0.062	17.116	1.000
CgC - CpC	0.213	0.489	0.435	17.470	1.000
CgC - CpS	-17.120	6.773	-2.528	9.039	.826
CgC - CpT	-1.582	1.024	-1.544	10.854	.999
CgC - SgC	-1.544	0.723	-2.136	12.985	.960
CgC - SgS	-21.717	0.987	-21.994	11.008	< .001
CgC - SgT	0.865	0.579	1.493	15.410	1.000
CgC - SpC	0.005	0.371	0.014	15.086	1.000
CgC - SpS	-10.149	0.831	-12.219	11.932	< .001
CgC - SpT	1.379	0.501	2.751	17.222	.751
CgC - TgC	0.018	0.488	0.037	17.499	1.000
CgC - TgS	-22.349	6.067	-3.684	9.049	.337
CgC - TgT	0.796	0.353	2.257	13.361	.935
CgC - TpC	0.048	0.403	0.119	17.177	1.000
CgC - TpS	-16.224	5.034	-3.223	9.071	.517
CgC - TpT	0.189	0.379	0.500	15.731	1.000
CgC - VEgC	0.897	0.531	1.689	16.525	.999
CgC - VEgS	-13.336	0.871	-15.312	11.641	< .001

Comparison	Mean Difference	SE	t	df	p_{tukey}
CgC - VEgT	-1.370	0.512	-2.678	16.986	.788
CgC - VEpC	-0.007	0.342	-0.019	12.141	1.000
CgC - VEpS	-15.291	6.150	-2.486	9.047	.842
CgC - VEpT	-1.834	0.392	-4.675	16.638	.057
CgC - VgC	2.332	0.377	6.178	15.638	.005
CgC - VgS	-17.477	0.670	-26.079	13.710	< .001
CgC - VgT	2.256	0.451	4.997	17.985	.029
CgC - VpC	1.864	0.634	2.938	14.304	.647
CgC - VpS	-19.241	7.243	-2.656	9.034	.774
CgC - VpT	2.113	0.346	6.101	12.648	.010
CgC - VEpTS	-5.638	0.486	-11.596	17.534	< .001
CgC - TpTS	-5.525	0.348	-15.864	12.871	< .001
CgC - BpTS	-3.249	0.457	-7.106	17.949	< .001
CgC - CpTS	-5.389	0.716	-7.530	13.076	.001
CgC - VpTS	-5.593	2.073	-2.699	9.424	.757
CgC - SpTS	-4.056	1.194	-3.398	10.335	.433
CgC - VEgTS	-5.748	0.440	-13.053	17.992	< .001
CgC - TgTS	-6.348	0.367	-17.282	14.779	< .001
CgC - BgTS	-3.292	0.436	-7.548	17.971	< .001
CgC - CgTS	-5.945	0.518	-11.477	16.840	< .001
CgC - VgTS	-4.876	0.625	-7.799	14.470	< .001
CgC - SgTS	-7.103	0.623	-11.396	14.507	< .001
CgS - CgT	14.781	0.961	15.378	12.540	< .001
CgS - CpC	15.026	0.953	15.773	12.194	< .001
CgS - CpS	-2.308	6.822	-0.338	9.301	1.000

Comparison	Mean Difference	SE	t	df	p_{tukey}
CgS - CpT	13.230	1.310	10.096	17.797	< .001
CgS - SgC	13.268	1.091	12.160	16.621	< .001
CgS - SgS	-6.904	1.282	-5.387	17.921	.014
CgS - SgT	15.677	1.002	15.652	14.068	< .001
CgS - SpC	14.818	0.897	16.512	9.902	< .001
CgS - SpS	4.664	1.165	4.002	17.703	.165
CgS - SpT	16.191	0.959	16.889	12.440	< .001
CgS - TgC	14.830	0.952	15.581	12.164	< .001
CgS - TgS	-7.537	6.122	-1.231	9.376	1.000
CgS - TgT	15.609	0.890	17.535	9.600	< .001
CgS - TpC	14.860	0.911	16.310	10.478	< .001
CgS - TpS	-1.411	5.100	-0.277	9.546	1.000
CgS - TpT	15.002	0.901	16.656	10.041	< .001
CgS - VEgC	15.710	0.975	16.117	13.070	< .001
CgS - VEgS	1.476	1.194	1.236	17.899	1.000
CgS - VEgT	13.442	0.964	13.941	12.659	< .001
CgS - VEpC	14.806	0.886	16.715	9.418	< .001
CgS - VEpS	-0.479	6.204	-0.077	9.366	1.000
CgS - VEpT	12.979	0.906	14.318	10.282	< .001
CgS - VgC	17.144	0.900	19.045	10.019	< .001
CgS - VgS	-2.664	1.057	-2.521	15.801	.856
CgS - VgT	17.068	0.934	18.282	11.415	< .001
CgS - VpC	16.676	1.035	16.119	15.160	< .001
CgS - VpS	-4.429	7.289	-0.608	9.264	1.000
CgS - VpT	16.925	0.888	19.070	9.491	< .001

Comparison	Mean Difference	SE	t	df	p_{tukey}
CgS - VEpTS	9.174	0.951	9.647	12.127	< .001
CgS - TpTS	9.287	0.888	10.455	9.525	< .001
CgS - BpTS	11.563	0.936	12.348	11.533	< .001
CgS - CpTS	9.424	1.086	8.675	16.517	< .001
CgS - VpTS	9.219	2.228	4.138	12.182	.173
CgS - SpTS	10.756	1.447	7.435	16.800	< .001
CgS - VEgTS	9.065	0.928	9.765	11.195	< .001
CgS - TgTS	8.465	0.896	9.448	9.842	< .001
CgS - BgTS	11.520	0.926	12.436	11.114	< .001
CgS - CgTS	8.867	0.968	9.165	12.791	< .001
CgS - VgTS	9.936	1.029	9.656	14.987	< .001
CgS - SgTS	7.709	1.028	7.501	14.949	< .001
CgT - CpC	0.244	0.546	0.448	17.945	1.000
CgT - CpS	-17.089	6.777	-2.522	9.062	.829
CgT - CpT	-1.551	1.052	-1.473	11.897	1.000
CgT - SgC	-1.513	0.762	-1.985	14.867	.984
CgT - SgS	-21.685	1.016	-21.335	12.128	< .001
CgT - SgT	0.896	0.627	1.428	17.298	1.000
CgT - SpC	0.037	0.442	0.083	13.163	1.000
CgT - SpS	-10.117	0.865	-11.698	13.470	< .001
CgT - SpT	1.410	0.556	2.535	17.996	.856
CgT - TgC	0.049	0.544	0.091	17.935	1.000
CgT - TgS	-22.318	6.072	-3.676	9.077	.339
CgT - TgT	0.828	0.427	1.937	11.854	.984
CgT - TpC	0.079	0.470	0.169	15.246	1.000

Comparison	Mean Difference	SE	t	df	p_{tukey}
CgT - TpS	-16.193	5.040	-3.213	9.112	.521
CgT - TpT	0.221	0.449	0.491	13.717	1.000
CgT - VEgC	0.929	0.583	1.591	17.895	1.000
CgT - VEgS	-13.305	0.904	-14.722	13.058	< .001
CgT - VEgT	-1.339	0.566	-2.367	17.994	.917
CgT - VEpC	0.025	0.418	0.059	11.016	1.000
CgT - VEpS	-15.260	6.155	-2.479	9.075	.845
CgT - VEpT	-1.802	0.460	-3.914	14.606	.210
CgT - VgC	2.363	0.448	5.275	13.633	.030
CgT - VgS	-17.445	0.712	-24.494	15.724	< .001
CgT - VgT	2.287	0.512	4.469	17.308	.078
CgT - VpC	1.895	0.679	2.792	16.349	.728
CgT - VpS	-19.210	7.247	-2.651	9.054	.776
CgT - VpT	2.144	0.422	5.081	11.358	.053
CgT - VEpTS	-5.607	0.543	-10.330	17.920	< .001
CgT - TpTS	-5.494	0.424	-12.967	11.511	< .001
CgT - BpTS	-3.218	0.517	-6.224	17.460	.003
CgT - CpTS	-5.357	0.755	-7.094	14.980	.001
CgT - VpTS	-5.562	2.087	-2.666	9.673	.772
CgT - SpTS	-4.025	1.218	-3.305	11.103	.469
CgT - VEgTS	-5.716	0.502	-11.385	16.967	< .001
CgT - TgTS	-6.316	0.439	-14.373	12.914	< .001
CgT - BgTS	-3.261	0.498	-6.542	16.820	.002
CgT - CgTS	-5.914	0.571	-10.349	17.975	< .001
CgT - VgTS	-4.845	0.670	-7.229	16.511	< .001

Comparison	Mean Difference	SE	t	df	p_{tukey}
CgT - SgTS	-7.072	0.668	-10.581	16.546	< .001
CpC - CpS	-17.333	6.776	-2.558	9.055	.815
CpC - CpT	-1.795	1.045	-1.719	11.607	.996
CpC - SgC	-1.758	0.751	-2.339	14.381	.916
CpC - SgS	-21.930	1.008	-21.750	11.818	< .001
CpC - SgT	0.651	0.614	1.061	16.909	1.000
CpC - SpC	-0.208	0.423	-0.491	13.586	1.000
CpC - SpS	-10.362	0.855	-12.114	13.055	< .001
CpC - SpT	1.166	0.541	2.154	17.971	.967
CpC - TgC	-0.195	0.529	-0.369	18.000	1.000
CpC - TgS	-22.562	6.071	-3.717	9.069	.326
CpC - TgT	0.583	0.408	1.431	12.168	1.000
CpC - TpC	-0.165	0.452	-0.365	15.743	1.000
CpC - TpS	-16.437	5.038	-3.262	9.100	.499
CpC - TpT	-0.024	0.430	-0.055	14.174	1.000
CpC - VEgC	0.684	0.569	1.202	17.694	1.000
CpC - VEgS	-13.549	0.895	-15.146	12.671	< .001
CpC - VEgT	-1.584	0.551	-2.874	17.904	.685
CpC - VEpC	-0.220	0.398	-0.552	11.245	1.000
CpC - VEpS	-15.504	6.154	-2.520	9.067	.830
CpC - VEpT	-2.047	0.442	-4.628	15.098	.070
CpC - VgC	2.119	0.429	4.937	14.086	.047
CpC - VgS	-17.690	0.701	-25.251	15.226	< .001
CpC - VgT	2.042	0.495	4.123	17.623	.137
CpC - VpC	1.651	0.666	2.477	15.867	.873

Comparison	Mean Difference	SE	t	df	p_{tukey}
CpC - VpS	-19.454	7.246	-2.685	9.048	.761
CpC - VpT	1.900	0.402	4.725	11.623	.083
CpC - VEpTS	-5.852	0.527	-11.095	17.998	< .001
CpC - TpTS	-5.738	0.404	-14.212	11.792	< .001
CpC - BpTS	-3.462	0.501	-6.914	17.737	< .001
CpC - CpTS	-5.602	0.744	-7.527	14.491	< .001
CpC - VpTS	-5.806	2.083	-2.788	9.603	.717
CpC - SpTS	-4.269	1.211	-3.525	10.888	.376
CpC - VEgTS	-5.961	0.485	-12.280	17.346	< .001
CpC - TgTS	-6.561	0.420	-15.611	13.319	< .001
CpC - BgTS	-3.505	0.482	-7.278	17.221	< .001
CpC - CgTS	-6.158	0.557	-11.060	17.847	< .001
CpC - VgTS	-5.089	0.658	-7.737	16.037	< .001
CpC - SgTS	-7.316	0.656	-11.154	16.074	< .001
CpS - CpT	15.538	6.836	2.273	9.374	.914
CpS - SgC	15.576	6.797	2.292	9.167	.908
CpS - SgS	-4.596	6.830	-0.673	9.344	1.000
CpS - SgT	17.985	6.783	2.651	9.093	.776
CpS - SpC	17.125	6.769	2.530	9.015	.825
CpS - SpS	6.971	6.809	1.024	9.232	1.000
CpS - SpT	18.499	6.777	2.730	9.060	.742
CpS - TgC	17.138	6.776	2.529	9.055	.826
CpS - TgS	-5.229	9.082	-0.576	17.785	1.000
CpS - TgT	17.917	6.768	2.647	9.010	.777
CpS - TpC	17.168	6.770	2.536	9.025	.823

Comparison	Mean Difference	SE	t	df	p_{tukey}
CpS - TpS	0.896	8.427	0.106	16.612	1.000
CpS - TpT	17.310	6.769	2.557	9.017	.815
CpS - VEgC	18.017	6.779	2.658	9.072	.773
CpS - VEgS	3.784	6.814	0.555	9.259	1.000
CpS - VEgT	15.750	6.778	2.324	9.064	.898
CpS - VEpC	17.114	6.767	2.529	9.007	.826
CpS - VEpS	1.829	9.138	0.200	17.834	1.000
CpS - VEpT	15.287	6.770	2.258	9.022	.916
CpS - VgC	19.452	6.769	2.874	9.017	.676
CpS - VgS	-0.357	6.792	-0.052	9.138	1.000
CpS - VgT	19.376	6.773	2.861	9.041	.682
CpS - VpC	18.984	6.788	2.797	9.119	.712
CpS - VpS	-2.121	9.907	-0.214	17.919	1.000
CpS - VpT	19.233	6.767	2.842	9.008	.691
CpS - VEpTS	11.482	6.776	1.695	9.054	.995
CpS - TpTS	11.595	6.767	1.713	9.009	.994
CpS - BpTS	13.871	6.774	2.048	9.043	.962
CpS - CpTS	11.732	6.796	1.726	9.162	.994
CpS - VpTS	11.527	7.069	1.631	10.637	.998
CpS - SpTS	13.064	6.863	1.904	9.521	.982
CpS - VEgTS	11.373	6.773	1.679	9.037	.995
CpS - TgTS	10.773	6.768	1.592	9.014	.998
CpS - BgTS	13.828	6.772	2.042	9.036	.963
CpS - CgTS	11.175	6.778	1.649	9.067	.996
CpS - VgTS	12.244	6.787	1.804	9.115	.989

Comparison	Mean Difference	SE	t	df	p_{tukey}
CpS - SgTS	10.017	6.787	1.476	9.114	.999
CpT - SgC	0.038	1.172	0.032	15.696	1.000
CpT - SgS	-20.134	1.351	-14.899	17.970	< .001
CpT - SgT	2.447	1.089	2.246	13.217	.937
CpT - SpC	1.587	0.994	1.596	9.728	.998
CpT - SpS	-8.567	1.242	-6.900	17.072	.001
CpT - SpT	2.961	1.050	2.820	11.813	.706
CpT - TgC	1.600	1.044	1.533	11.582	.999
CpT - TgS	-20.767	6.137	-3.384	9.466	.445
CpT - TgT	2.378	0.988	2.408	9.484	.873
CpT - TpC	1.630	1.007	1.619	10.195	.998
CpT - TpS	-14.642	5.118	-2.861	9.677	.683
CpT - TpT	1.771	0.997	1.776	9.840	.992
CpT - VEgC	2.479	1.065	2.328	12.347	.912
CpT - VEgS	-11.754	1.269	-9.263	17.431	< .001
CpT - VEgT	0.212	1.055	0.201	11.998	1.000
CpT - VEpC	1.575	0.984	1.601	9.338	.998
CpT - VEpS	-13.709	6.219	-2.204	9.453	.932
CpT - VEpT	-0.251	1.003	-0.251	10.037	1.000
CpT - VgC	3.914	0.997	3.926	9.823	.253
CpT - VgS	-15.895	1.140	-13.938	14.839	< .001
CpT - VgT	3.838	1.027	3.736	10.961	.298
CpT - VpC	3.446	1.120	3.077	14.217	.572
CpT - VpS	-17.659	7.302	-2.418	9.327	.868
CpT - VpT	3.695	0.986	3.749	9.397	.311

Comparison	Mean Difference	SE	t	df	p_{tukey}
CpT - VEpTS	-4.056	1.043	-3.889	11.550	.244
CpT - TpTS	-3.943	0.986	-3.998	9.423	.239
CpT - BpTS	-1.667	1.030	-1.619	11.058	.998
CpT - CpTS	-3.807	1.168	-3.260	15.582	.472
CpT - VpTS	-4.011	2.269	-1.768	12.877	.995
CpT - SpTS	-2.474	1.509	-1.640	17.522	.999
CpT - VEgTS	-4.165	1.022	-4.074	10.780	.203
CpT - TgTS	-4.765	0.993	-4.798	9.680	.094
CpT - BgTS	-1.710	1.021	-1.676	10.714	.997
CpT - CgTS	-4.363	1.058	-4.123	12.109	.177
CpT - VgTS	-3.294	1.115	-2.955	14.054	.638
CpT - SgTS	-5.521	1.114	-4.958	14.018	.046
SgC - SgS	-20.172	1.140	-17.693	16.057	< .001
SgC - SgT	2.409	0.813	2.965	16.658	.633
SgC - SpC	1.550	0.680	2.279	10.623	.919
SgC - SpS	-8.604	1.007	-8.541	17.525	< .001
SgC - SpT	2.923	0.759	3.851	14.729	.228
SgC - TgC	1.562	0.750	2.082	14.337	.972
SgC - TgS	-20.805	6.094	-3.414	9.208	.435
SgC - TgT	2.341	0.670	3.492	10.082	.396
SgC - TpC	1.592	0.698	2.281	11.634	.922
SgC - TpS	-14.679	5.066	-2.897	9.302	.665
SgC - TpT	1.734	0.684	2.533	10.868	.833
SgC - VEgC	2.442	0.779	3.134	15.557	.540
SgC - VEgS	-11.792	1.041	-11.328	17.186	< .001

Comparison	Mean Difference	SE	t	df	p_{tukey}
SgC - VEgT	0.174	0.766	0.227	15.028	1.000
SgC - VEpC	1.538	0.665	2.314	9.756	.904
SgC - VEpS	-13.747	6.177	-2.226	9.202	.926
SgC - VEpT	-0.289	0.692	-0.418	11.294	1.000
SgC - VgC	3.876	0.684	5.670	10.830	.027
SgC - VgS	-15.932	0.880	-18.110	17.838	< .001
SgC - VgT	3.800	0.727	5.227	13.194	.034
SgC - VpC	3.408	0.853	3.996	17.521	.168
SgC - VpS	-17.697	7.266	-2.436	9.146	.861
SgC - VpT	3.657	0.667	5.484	9.888	.041
SgC - VEpTS	-4.094	0.749	-5.464	14.283	.020
SgC - TpTS	-3.981	0.668	-5.959	9.947	.023
SgC - BpTS	-1.705	0.731	-2.333	13.381	.914
SgC - CpTS	-3.844	0.915	-4.202	17.997	.118
SgC - VpTS	-4.049	2.150	-1.884	10.799	.987
SgC - SpTS	-2.512	1.323	-1.899	14.219	.990
SgC - VEgTS	-4.203	0.720	-5.835	12.839	.015
SgC - TgTS	-4.803	0.678	-7.083	10.516	.005
SgC - BgTS	-1.748	0.718	-2.435	12.707	.878
SgC - CgTS	-4.401	0.770	-5.714	15.203	.012
SgC - VgTS	-3.332	0.846	-3.938	17.410	.185
SgC - SgTS	-5.559	0.845	-6.581	17.384	.002
SgS - SgT	22.581	1.055	21.410	13.528	< .001
SgS - SpC	21.722	0.956	22.715	9.790	< .001
SgS - SpS	11.568	1.211	9.551	17.345	< .001

Comparison	Mean Difference	SE	t	df	p_{tukey}
SgS - SpT	23.096	1.014	22.775	12.038	< .001
SgS - TgC	21.735	1.008	21.571	11.791	< .001
SgS - TgS	-0.633	6.131	-0.103	9.429	1.000
SgS - TgT	22.513	0.949	23.710	9.525	< .001
SgS - TpC	21.765	0.969	22.456	10.296	< .001
SgS - TpS	5.493	5.111	1.075	9.624	1.000
SgS - TpT	21.906	0.959	22.833	9.912	< .001
SgS - VEgC	22.614	1.029	21.972	12.608	< .001
SgS - VEgS	8.380	1.239	6.763	17.650	.001
SgS - VEgT	20.346	1.019	19.962	12.236	< .001
SgS - VEpC	21.710	0.945	22.964	9.366	< .001
SgS - VEpS	6.425	6.213	1.034	9.418	1.000
SgS - VEpT	19.883	0.965	20.608	10.124	< .001
SgS - VgC	24.049	0.959	25.079	9.893	< .001
SgS - VgS	4.240	1.107	3.829	15.204	.232
SgS - VgT	23.972	0.990	24.206	11.123	< .001
SgS - VpC	23.580	1.086	21.712	14.569	< .001
SgS - VpS	2.476	7.297	0.339	9.301	1.000
SgS - VpT	23.829	0.947	25.161	9.430	< .001
SgS - VEpTS	16.078	1.007	15.971	11.757	< .001
SgS - TpTS	16.191	0.948	17.083	9.459	< .001
SgS - BpTS	18.467	0.993	18.597	11.228	< .001
SgS - CpTS	16.328	1.135	14.380	15.946	< .001
SgS - VpTS	16.123	2.252	7.159	12.600	.002
SgS - SpTS	17.660	1.484	11.900	17.278	< .001

Comparison	Mean Difference	SE	t	df	p_{tukey}
SgS - VEgTS	15.969	0.985	16.207	10.928	< .001
SgS - TgTS	15.369	0.955	16.094	9.738	< .001
SgS - BgTS	18.424	0.984	18.733	10.857	< .001
SgS - CgTS	15.771	1.022	15.426	12.355	< .001
SgS - VgTS	16.840	1.081	15.582	14.401	< .001
SgS - SgTS	14.613	1.080	13.536	14.364	< .001
SgT - SpC	-0.859	0.524	-1.639	11.857	.998
SgT - SpS	-11.013	0.910	-12.108	15.208	< .001
SgT - SpT	0.514	0.624	0.825	17.195	1.000
SgT - TgC	-0.847	0.613	-1.381	16.871	1.000
SgT - TgS	-23.214	6.078	-3.819	9.116	.293
SgT - TgT	-0.068	0.512	-0.133	10.924	1.000
SgT - TpC	-0.817	0.548	-1.491	13.503	1.000
SgT - TpS	-17.088	5.048	-3.385	9.169	.447
SgT - TpT	-0.675	0.530	-1.274	12.270	1.000
SgT - VEgC	0.033	0.648	0.050	17.716	1.000
SgT - VEgS	-14.201	0.947	-15.002	14.717	< .001
SgT - VEgT	-2.235	0.632	-3.537	17.410	.330
SgT - VEpC	-0.871	0.504	-1.728	10.350	.995
SgT - VEpS	-16.156	6.161	-2.622	9.113	.788
SgT - VEpT	-2.698	0.540	-4.999	12.967	.049
SgT - VgC	1.467	0.529	2.773	12.207	.730
SgT - VgS	-18.341	0.766	-23.948	17.348	< .001
SgT - VgT	1.391	0.584	2.381	15.668	.907
SgT - VpC	0.999	0.735	1.360	17.726	1.000

Comparison	Mean Difference	SE	t	df	p_{tukey}
SgT - VpS	-20.106	7.253	-2.772	9.081	.723
SgT - VpT	1.248	0.507	2.460	10.583	.860
SgT - VEpTS	-6.503	0.611	-10.635	16.822	< .001
SgT - TpTS	-6.390	0.509	-12.560	10.688	< .001
SgT - BpTS	-4.114	0.589	-6.988	15.889	.001
SgT - CpTS	-6.253	0.806	-7.758	16.759	< .001
SgT - VpTS	-6.458	2.106	-3.067	10.011	.585
SgT - SpTS	-4.921	1.250	-3.936	12.110	.225
SgT - VEgTS	-6.612	0.576	-11.486	15.224	< .001
SgT - TgTS	-7.212	0.522	-13.818	11.676	< .001
SgT - BgTS	-4.157	0.573	-7.261	15.050	.001
SgT - CgTS	-6.810	0.637	-10.690	17.522	< .001
SgT - VgTS	-5.741	0.727	-7.897	17.803	< .001
SgT - SgTS	-7.968	0.725	-10.986	17.818	< .001
SpC - SpS	-10.154	0.793	-12.798	10.168	< .001
SpC - SpT	1.374	0.437	3.145	13.278	.538
SpC - TgC	0.013	0.422	0.030	13.626	1.000
SpC - TgS	-22.354	6.062	-3.688	9.019	.336
SpC - TgT	0.791	0.253	3.125	17.294	.541
SpC - TpC	0.043	0.319	0.134	16.988	1.000
SpC - TpS	-16.229	5.028	-3.228	9.027	.515
SpC - TpT	0.184	0.288	0.639	17.908	1.000
SpC - VEgC	0.892	0.471	1.894	12.624	.989
SpC - VEgS	-13.341	0.836	-15.967	10.048	< .001
SpC - VEgT	-1.376	0.449	-3.065	13.031	.580

Comparison	Mean Difference	SE	t	df	p_{tukey}
SpC - VEpC	-0.012	0.237	-0.050	15.861	1.000
SpC - VEpS	-15.297	6.145	-2.489	9.018	.841
SpC - VEpT	-1.839	0.306	-6.015	17.463	.005
SpC - VgC	2.327	0.286	8.122	17.933	< .001
SpC - VgS	-17.482	0.623	-28.042	10.957	< .001
SpC - VgT	2.250	0.379	5.945	14.830	.009
SpC - VpC	1.859	0.585	3.178	11.248	.528
SpC - VpS	-19.246	7.239	-2.659	9.013	.773
SpC - VpT	2.108	0.244	8.642	16.555	< .001
SpC - VEpTS	-5.644	0.420	-13.452	13.677	< .001
SpC - TpTS	-5.530	0.247	-22.412	16.816	< .001
SpC - BpTS	-3.254	0.386	-8.441	14.610	< .001
SpC - CpTS	-5.394	0.672	-8.025	10.664	.002
SpC - VpTS	-5.598	2.058	-2.720	9.165	.746
SpC - SpTS	-4.061	1.168	-3.476	9.522	.407
SpC - VEgTS	-5.753	0.365	-15.747	15.272	< .001
SpC - TgTS	-6.353	0.273	-23.277	17.979	< .001
SpC - BgTS	-3.297	0.360	-9.151	15.446	< .001
SpC - CgTS	-5.950	0.456	-13.050	12.893	< .001
SpC - VgTS	-4.882	0.575	-8.490	11.334	< .001
SpC - SgTS	-7.108	0.573	-12.410	11.353	< .001
SpS - SpT	11.528	0.862	13.371	13.351	< .001
SpS - TgC	10.167	0.855	11.898	13.018	< .001
SpS - TgS	-12.200	6.108	-1.998	9.290	.971
SpS - TgT	10.945	0.785	13.940	9.778	< .001

Comparison	Mean Difference	SE	t	df	p_{tukey}
SpS - TpC	10.197	0.809	12.605	10.909	< .001
SpS - TpS	-6.075	5.083	-1.195	9.421	1.000
SpS - TpT	10.338	0.797	12.969	10.347	< .001
SpS - VEgC	11.046	0.880	12.554	14.092	< .001
SpS - VEgS	-3.187	1.118	-2.850	17.946	.698
SpS - VEgT	8.778	0.868	10.110	13.612	< .001
SpS - VEpC	10.142	0.780	12.999	9.543	< .001
SpS - VEpS	-5.143	6.190	-0.831	9.282	1.000
SpS - VEpT	8.315	0.804	10.347	10.658	< .001
SpS - VgC	12.481	0.797	15.668	10.320	< .001
SpS - VgS	-7.328	0.970	-7.554	16.894	< .001
SpS - VgT	12.404	0.834	14.871	12.094	< .001
SpS - VpC	12.013	0.946	12.702	16.315	< .001
SpS - VpS	-9.092	7.277	-1.249	9.203	1.000
SpS - VpT	12.262	0.782	15.675	9.637	< .001
SpS - VEpTS	4.510	0.853	5.285	12.972	.032
SpS - TpTS	4.624	0.783	5.904	9.680	.026
SpS - BpTS	6.900	0.837	8.240	12.242	< .001
SpS - CpTS	4.760	1.002	4.750	17.453	.047
SpS - VpTS	4.556	2.188	2.082	11.485	.965
SpS - SpTS	6.093	1.385	4.400	15.692	.096
SpS - VEgTS	4.401	0.828	5.314	11.819	.036
SpS - TgTS	3.801	0.792	4.801	10.091	.089
SpS - BgTS	6.857	0.826	8.301	11.717	< .001
SpS - CgTS	4.204	0.872	4.821	13.767	.058

Comparison	Mean Difference	SE	t	df	p_{tukey}
SpS - VgTS	5.273	0.940	5.611	16.149	.012
SpS - SgTS	3.046	0.938	3.246	16.112	.478
SpT - TgC	-1.361	0.540	-2.521	17.964	.862
SpT - TgS	-23.728	6.072	-3.908	9.075	.268
SpT - TgT	-0.583	0.422	-1.381	11.939	1.000
SpT - TpC	-1.331	0.464	-2.866	15.385	.687
SpT - TpS	-17.603	5.039	-3.493	9.109	.405
SpT - TpT	-1.190	0.444	-2.682	13.842	.778
SpT - VEgC	-0.482	0.579	-0.832	17.848	1.000
SpT - VEgS	-14.715	0.901	-16.330	12.946	< .001
SpT - VEgT	-2.749	0.561	-4.897	17.980	.035
SpT - VEpC	-1.386	0.413	-3.359	11.078	.445
SpT - VEpS	-16.670	6.155	-2.709	9.073	.751
SpT - VEpT	-3.213	0.455	-7.057	14.742	.002
SpT - VgC	0.953	0.443	2.153	13.758	.959
SpT - VgS	-18.856	0.709	-26.600	15.585	< .001
SpT - VgT	0.877	0.507	1.729	17.405	.998
SpT - VpC	0.485	0.675	0.718	16.216	1.000
SpT - VpS	-20.620	7.247	-2.845	9.052	.689
SpT - VpT	0.734	0.416	1.763	11.430	.995
SpT - VEpTS	-7.017	0.538	-13.035	17.953	< .001
SpT - TpTS	-6.904	0.418	-16.518	11.587	< .001
SpT - BpTS	-4.628	0.512	-9.034	17.546	< .001
SpT - CpTS	-6.768	0.752	-8.999	14.842	< .001
SpT - VpTS	-6.972	2.085	-3.343	9.652	.461

Comparison	Mean Difference	SE	t	df	p_{tukey}
SpT - SpTS	-5.435	1.216	-4.470	11.040	.122
SpT - VEgTS	-7.126	0.497	-14.332	17.079	< .001
SpT - TgTS	-7.726	0.434	-17.806	13.025	< .001
SpT - BgTS	-4.671	0.494	-9.463	16.938	< .001
SpT - CgTS	-7.324	0.567	-12.913	17.950	< .001
SpT - VgTS	-6.255	0.667	-9.384	16.381	< .001
SpT - SgTS	-8.482	0.665	-12.760	16.417	< .001
TgC - TgS	-22.367	6.070	-3.685	9.068	.336
TgC - TgT	0.778	0.406	1.918	12.198	.986
TgC - TpC	0.030	0.450	0.067	15.789	1.000
TgC - TpS	-16.242	5.038	-3.224	9.099	.516
TgC - TpT	0.171	0.429	0.400	14.217	1.000
TgC - VEgC	0.879	0.568	1.548	17.671	1.000
TgC - VEgS	-13.354	0.894	-14.941	12.637	< .001
TgC - VEgT	-1.388	0.550	-2.526	17.890	.859
TgC - VEpC	-0.025	0.396	-0.062	11.268	1.000
TgC - VEpS	-15.309	6.154	-2.488	9.066	.842
TgC - VEpT	-1.851	0.441	-4.202	15.143	.135
TgC - VgC	2.314	0.428	5.412	14.129	.022
TgC - VgS	-17.495	0.700	-25.008	15.180	< .001
TgC - VgT	2.238	0.494	4.530	17.648	.069
TgC - VpC	1.846	0.665	2.774	15.822	.736
TgC - VpS	-19.259	7.246	-2.658	9.048	.773
TgC - VpT	2.095	0.400	5.234	11.649	.042
TgC - VEpTS	-5.656	0.526	-10.753	17.999	< .001

Comparison	Mean Difference	SE	t	df	p_{tukey}
TgC - TpTS	-5.543	0.402	-13.789	11.819	< .001
TgC - BpTS	-3.267	0.499	-6.543	17.758	.002
TgC - CpTS	-5.407	0.743	-7.274	14.447	.001
TgC - VpTS	-5.611	2.082	-2.695	9.597	.759
TgC - SpTS	-4.074	1.211	-3.365	10.869	.443
TgC - VEgTS	-5.765	0.484	-11.914	17.378	< .001
TgC - TgTS	-6.365	0.419	-15.208	13.358	< .001
TgC - BgTS	-3.310	0.480	-6.894	17.255	.001
TgC - CgTS	-5.963	0.556	-10.734	17.830	< .001
TgC - VgTS	-4.894	0.657	-7.453	15.992	< .001
TgC - SgTS	-7.121	0.655	-10.875	16.030	< .001
TgS - TgT	23.146	6.061	3.819	9.013	.294
TgS - TpC	22.397	6.064	3.693	9.031	.334
TgS - TpS	6.125	7.871	0.778	17.404	1.000
TgS - TpT	22.538	6.063	3.718	9.022	.326
TgS - VEgC	23.246	6.074	3.827	9.090	.291
TgS - VEgS	9.013	6.113	1.474	9.323	.999
TgS - VEgT	20.979	6.072	3.455	9.080	.420
TgS - VEpC	22.343	6.060	3.687	9.009	.336
TgS - VEpS	7.058	8.628	0.818	17.997	1.000
TgS - VEpT	20.516	6.063	3.383	9.027	.449
TgS - VgC	24.681	6.063	4.071	9.021	.226
TgS - VgS	4.872	6.088	0.800	9.172	1.000
TgS - VgT	24.605	6.068	4.055	9.051	.230
TgS - VpC	24.213	6.084	3.980	9.149	.247

Comparison	Mean Difference	SE	t	df	p_{tukey}
TgS - VpS	3.108	9.438	0.329	17.461	1.000
TgS - VpT	24.462	6.061	4.036	9.010	.235
TgS - VEpTS	16.711	6.070	2.753	9.067	.731
TgS - TpTS	16.824	6.061	2.776	9.011	.721
TgS - BpTS	19.100	6.068	3.148	9.054	.550
TgS - CpTS	16.961	6.093	2.784	9.203	.718
TgS - VpTS	16.756	6.396	2.620	11.031	.797
TgS - SpTS	18.293	6.167	2.966	9.649	.633
TgS - VEgTS	16.602	6.067	2.736	9.047	.739
TgS - TgTS	16.002	6.062	2.640	9.018	.781
TgS - BgTS	19.057	6.066	3.141	9.045	.553
TgS - CgTS	16.404	6.073	2.701	9.083	.754
TgS - VgTS	17.473	6.083	2.872	9.143	.677
TgS - SgTS	15.246	6.083	2.506	9.142	.835
TgT - TpC	-0.748	0.298	-2.509	15.245	.859
TgT - TpS	-17.020	5.027	-3.386	9.018	.448
TgT - TpT	-0.607	0.265	-2.294	16.779	.935
TgT - VEgC	0.101	0.457	0.220	11.464	1.000
TgT - VEgS	-14.133	0.828	-17.073	9.697	< .001
TgT - VEgT	-2.167	0.434	-4.991	11.758	.057
TgT - VEpC	-0.803	0.208	-3.855	17.444	.209
TgT - VEpS	-16.088	6.144	-2.618	9.012	.790
TgT - VEpT	-2.630	0.284	-9.270	15.889	< .001
TgT - VgC	1.536	0.263	5.840	16.859	.007
TgT - VgS	-18.273	0.613	-29.812	10.308	< .001

Comparison	Mean Difference	SE	t	df	p_{tukey}
TgT - VgT	1.459	0.361	4.041	13.146	.187
TgT - VpC	1.067	0.574	1.861	10.506	.988
TgT - VpS	-20.037	7.238	-2.768	9.009	.724
TgT - VpT	1.316	0.216	6.102	17.824	.004
TgT - VEpTS	-6.435	0.404	-15.936	12.236	< .001
TgT - TpTS	-6.322	0.219	-28.873	17.920	< .001
TgT - BpTS	-4.046	0.368	-10.983	12.966	< .001
TgT - CpTS	-6.185	0.662	-9.337	10.110	< .001
TgT - VpTS	-6.390	2.055	-3.110	9.110	.567
TgT - SpTS	-4.853	1.163	-4.174	9.347	.198
TgT - VEgTS	-6.544	0.347	-18.851	13.521	< .001
TgT - TgTS	-7.144	0.248	-28.799	17.501	< .001
TgT - BgTS	-4.089	0.342	-11.958	13.674	< .001
TgT - CgTS	-6.742	0.442	-15.268	11.657	< .001
TgT - VgTS	-5.673	0.564	-10.066	10.564	< .001
TgT - SgTS	-7.900	0.561	-14.072	10.577	< .001
TpC - TpS	-16.272	5.031	-3.235	9.045	.512
TpC - TpT	0.141	0.328	0.430	17.462	1.000
TpC - VEgC	0.849	0.497	1.710	14.561	.998
TpC - VEgS	-13.384	0.850	-15.740	10.714	< .001
TpC - VEgT	-1.418	0.476	-2.982	15.083	.623
TpC - VEpC	-0.055	0.285	-0.192	13.691	1.000
TpC - VEpS	-15.339	6.147	-2.495	9.030	.839
TpC - VEpT	-1.882	0.344	-5.470	17.908	.012
TpC - VgC	2.284	0.327	6.984	17.401	< .001

Comparison	Mean Difference	SE	t	df	p_{tukey}
TpC - VgS	-17.525	0.643	-27.252	12.156	< .001
TpC - VgT	2.208	0.410	5.383	16.973	.016
TpC - VpC	1.816	0.606	2.998	12.603	.616
TpC - VpS	-19.289	7.241	-2.664	9.022	.770
TpC - VpT	2.065	0.290	7.109	14.363	.002
TpC - VEpTS	-5.686	0.448	-12.688	15.845	< .001
TpC - TpTS	-5.573	0.293	-19.031	14.647	< .001
TpC - BpTS	-3.297	0.417	-7.915	16.783	< .001
TpC - CpTS	-5.437	0.690	-7.875	11.698	.001
TpC - VpTS	-5.641	2.064	-2.733	9.272	.741
TpC - SpTS	-4.104	1.179	-3.482	9.858	.402
TpC - VEgTS	-5.796	0.398	-14.565	17.313	< .001
TpC - TgTS	-6.395	0.315	-20.289	16.724	< .001
TpC - BgTS	-3.340	0.393	-8.492	17.432	< .001
TpC - CgTS	-5.993	0.482	-12.422	14.910	< .001
TpC - VgTS	-4.924	0.596	-8.260	12.732	< .001
TpC - SgTS	-7.151	0.594	-12.037	12.761	< .001
TpS - TpT	16.413	5.029	3.264	9.032	.499
TpS - VEgC	17.121	5.043	3.395	9.131	.443
TpS - VEgS	2.888	5.090	0.567	9.470	1.000
TpS - VEgT	14.853	5.040	2.947	9.116	.642
TpS - VEpC	16.217	5.026	3.227	9.013	.515
TpS - VEpS	0.933	7.935	0.118	17.319	1.000
TpS - VEpT	14.390	5.030	2.861	9.039	.682
TpS - VgC	18.556	5.029	3.690	9.031	.335

Comparison	Mean Difference	SE	t	df	p_{tukey}
TpS - VgS	-1.253	5.059	-0.248	9.250	1.000
TpS - VgT	18.479	5.035	3.670	9.075	.341
TpS - VpC	18.088	5.054	3.579	9.216	.372
TpS - VpS	-3.017	8.810	-0.342	16.041	1.000
TpS - VpT	18.337	5.026	3.648	9.015	.349
TpS - VEpTS	10.585	5.038	2.101	9.098	.953
TpS - TpTS	10.699	5.027	2.128	9.016	.947
TpS - BpTS	12.975	5.035	2.577	9.079	.807
TpS - CpTS	10.835	5.065	2.139	9.295	.946
TpS - VpTS	10.631	5.426	1.959	11.912	.982
TpS - SpTS	12.168	5.155	2.361	9.943	.891
TpS - VEgTS	10.476	5.034	2.081	9.068	.956
TpS - TgTS	9.876	5.028	1.964	9.026	.974
TpS - BgTS	12.932	5.033	2.569	9.065	.810
TpS - CgTS	10.279	5.041	2.039	9.121	.964
TpS - VgTS	11.348	5.053	2.246	9.208	.921
TpS - SgTS	9.121	5.053	1.805	9.206	.990
TpT - VEgC	0.708	0.477	1.483	13.125	1.000
TpT - VEgS	-13.525	0.839	-16.119	10.208	< .001
TpT - VEgT	-1.560	0.455	-3.425	13.573	.400
TpT - VEpC	-0.196	0.250	-0.785	15.217	1.000
TpT - VEpS	-15.481	6.146	-2.519	9.021	.830
TpT - VEpT	-2.023	0.315	-6.416	17.804	.002
TpT - VgC	2.143	0.297	7.222	17.998	< .001
TpT - VgS	-17.666	0.628	-28.122	11.250	< .001

Comparison	Mean Difference	SE	t	df	p_{tukey}
TpT - VgT	2.066	0.386	5.348	15.474	.021
TpT - VpC	1.675	0.590	2.839	11.582	.696
TpT - VpS	-19.430	7.240	-2.684	9.015	.762
TpT - VpT	1.923	0.256	7.520	15.940	< .001
TpT - VEpTS	-5.828	0.427	-13.662	14.271	< .001
TpT - TpTS	-5.714	0.259	-22.104	16.225	< .001
TpT - BpTS	-3.438	0.393	-8.745	15.250	< .001
TpT - CpTS	-5.578	0.677	-8.245	10.915	.001
TpT - VpTS	-5.783	2.059	-2.808	9.191	.707
TpT - SpTS	-4.245	1.171	-3.626	9.603	.350
TpT - VEgTS	-5.937	0.373	-15.901	15.915	< .001
TpT - TgTS	-6.537	0.284	-23.049	17.801	< .001
TpT - BgTS	-3.482	0.369	-9.448	16.085	< .001
TpT - CgTS	-6.134	0.462	-13.265	13.421	< .001
TpT - VgTS	-5.066	0.580	-8.732	11.679	< .001
TpT - SgTS	-7.292	0.578	-12.617	11.701	< .001
VEgC - VEgS	-14.233	0.918	-15.503	13.643	< .001
VEgC - VEgT	-2.268	0.588	-3.854	17.938	.207
VEgC - VEpC	-0.904	0.448	-2.015	10.735	.973
VEgC - VEpS	-16.189	6.157	-2.629	9.087	.785
VEgC - VEpT	-2.731	0.488	-5.595	13.949	.017
VEgC - VgC	1.435	0.476	3.013	13.049	.608
VEgC - VgS	-18.374	0.730	-25.158	16.395	< .001
VEgC - VgT	1.358	0.537	2.531	16.756	.854
VEgC - VpC	0.967	0.698	1.386	16.965	1.000

Comparison	Mean Difference	SE	t	df	p_{tukey}
VEgC - VpS	-20.138	7.249	-2.778	9.063	.720
VEgC - VpT	1.216	0.452	2.690	11.031	.766
VEgC - VEpTS	-6.536	0.566	-11.540	17.640	< .001
VEgC - TpTS	-6.422	0.453	-14.163	11.164	< .001
VEgC - BpTS	-4.146	0.542	-7.655	16.947	< .001
VEgC - CpTS	-6.286	0.772	-8.139	15.671	< .001
VEgC - VpTS	-6.490	2.093	-3.101	9.785	.569
VEgC - SpTS	-4.953	1.229	-4.032	11.441	.206
VEgC - VEgTS	-6.645	0.527	-12.598	16.351	< .001
VEgC - TgTS	-7.245	0.468	-15.473	12.401	< .001
VEgC - BgTS	-4.189	0.524	-7.995	16.186	< .001
VEgC - CgTS	-6.842	0.594	-11.522	17.972	< .001
VEgC - VgTS	-5.773	0.689	-8.374	17.104	< .001
VEgC - SgTS	-8.000	0.688	-11.635	17.134	< .001
VEgS - VEgT	11.966	0.907	13.193	13.190	< .001
VEgS - VEpC	13.329	0.823	16.195	9.486	< .001
VEgS - VEpS	-1.955	6.196	-0.316	9.315	1.000
VEgS - VEpT	11.503	0.845	13.608	10.488	< .001
VEgS - VgC	15.668	0.839	18.685	10.183	< .001
VEgS - VgS	-4.141	1.005	-4.121	16.454	.144
VEgS - VgT	15.592	0.874	17.833	11.789	< .001
VEgS - VpC	15.200	0.981	15.488	15.835	< .001
VEgS - VpS	-5.905	7.282	-0.811	9.227	1.000
VEgS - VpT	15.449	0.825	18.727	9.571	< .001
VEgS - VEpTS	7.698	0.893	8.622	12.595	< .001

Comparison	Mean Difference	SE	t	df	p_{tukey}
VEgS - TpTS	7.811	0.826	9.458	9.610	< .001
VEgS - BpTS	10.087	0.877	11.497	11.924	< .001
VEgS - CpTS	7.948	1.036	7.673	17.098	< .001
VEgS - VpTS	7.743	2.204	3.513	11.761	.374
VEgS - SpTS	9.280	1.409	6.585	16.177	.002
VEgS - VEgTS	7.589	0.869	8.736	11.538	< .001
VEgS - TgTS	6.989	0.834	8.379	9.978	.002
VEgS - BgTS	10.044	0.867	11.590	11.445	< .001
VEgS - CgTS	7.391	0.910	8.118	13.336	< .001
VEgS - VgTS	8.460	0.976	8.672	15.663	< .001
VEgS - SgTS	6.233	0.974	6.398	15.625	.004
VEgT - VEpC	1.364	0.425	3.208	10.946	.515
VEgT - VEpS	-13.921	6.156	-2.262	9.078	.916
VEgT - VEpT	-0.463	0.467	-0.992	14.448	1.000
VEgT - VgC	3.702	0.454	8.148	13.491	< .001
VEgT - VgS	-16.106	0.716	-22.486	15.885	< .001
VEgT - VgT	3.626	0.517	7.008	17.189	< .001
VEgT - VpC	3.234	0.683	4.736	16.501	.052
VEgT - VpS	-17.871	7.248	-2.466	9.056	.850
VEgT - VpT	3.483	0.429	8.123	11.277	.001
VEgT - VEpTS	-4.268	0.548	-7.787	17.872	< .001
VEgT - TpTS	-4.155	0.430	-9.652	11.425	< .001
VEgT - BpTS	-1.879	0.523	-3.596	17.351	.305
VEgT - CpTS	-4.018	0.759	-5.294	15.142	.024
VEgT - VpTS	-4.223	2.088	-2.022	9.698	.968

Comparison	Mean Difference	SE	t	df	p_{tukey}
VEgT - SpTS	-2.686	1.220	-2.201	11.178	.941
VEgT - VEgTS	-4.377	0.508	-8.620	16.830	< .001
VEgT - TgTS	-4.977	0.446	-11.160	12.788	< .001
VEgT - BgTS	-1.922	0.504	-3.812	16.678	.228
VEgT - CgTS	-4.575	0.576	-7.936	17.993	< .001
VEgT - VgTS	-3.506	0.675	-5.198	16.658	.023
VEgT - SgTS	-5.733	0.673	-8.522	16.692	< .001
VEpC - VEpS	-15.285	6.144	-2.488	9.009	.842
VEpC - VEpT	-1.827	0.270	-6.774	14.287	.003
VEpC - VgC	2.339	0.248	9.439	15.310	< .001
VEpC - VgS	-17.470	0.607	-28.800	9.914	< .001
VEpC - VgT	2.262	0.350	6.460	11.976	.007
VEpC - VpC	1.871	0.567	3.300	10.054	.477
VEpC - VpS	-19.234	7.238	-2.658	9.006	.773
VEpC - VpT	2.119	0.197	10.762	17.884	< .001
VEpC - VEpTS	-5.632	0.394	-14.291	11.296	< .001
VEpC - TpTS	-5.519	0.200	-27.527	17.773	< .001
VEpC - BpTS	-3.243	0.358	-9.065	11.840	< .001
VEpC - CpTS	-5.382	0.657	-8.197	9.775	.002
VEpC - VpTS	-5.587	2.053	-2.721	9.076	.745
VEpC - SpTS	-4.050	1.159	-3.493	9.242	.404
VEpC - VEgTS	-5.741	0.336	-17.097	12.265	< .001
VEpC - TgTS	-6.341	0.232	-27.341	16.163	< .001
VEpC - BgTS	-3.286	0.330	-9.945	12.385	< .001
VEpC - CgTS	-5.938	0.433	-13.725	10.874	< .001

Comparison	Mean Difference	SE	t	df	p_{tukey}
VEpC - VgTS	-4.870	0.557	-8.748	10.095	.001
VEpC - SgTS	-7.096	0.554	-12.800	10.104	< .001
VEpS - VEpT	13.458	6.147	2.189	9.026	.934
VEpS - VgC	17.623	6.146	2.868	9.021	.679
VEpS - VgS	-2.185	6.171	-0.354	9.167	1.000
VEpS - VgT	17.547	6.151	2.853	9.050	.686
VEpS - VpC	17.155	6.167	2.782	9.145	.718
VEpS - VpS	-3.950	9.492	-0.416	17.537	1.000
VEpS - VpT	17.404	6.144	2.833	9.010	.695
VEpS - VEpTS	9.653	6.153	1.569	9.066	.998
VEpS - TpTS	9.766	6.144	1.590	9.011	.998
VEpS - BpTS	12.042	6.151	1.958	9.053	.975
VEpS - CpTS	9.903	6.176	1.603	9.197	.998
VEpS - VpTS	9.698	6.475	1.498	10.978	.999
VEpS - SpTS	11.235	6.249	1.798	9.632	.991
VEpS - VEgTS	9.544	6.150	1.552	9.045	.998
VEpS - TgTS	8.944	6.145	1.455	9.017	.999
VEpS - BgTS	11.999	6.150	1.951	9.044	.976
VEpS - CgTS	9.346	6.156	1.518	9.081	.999
VEpS - VgTS	10.415	6.166	1.689	9.139	.995
VEpS - SgTS	8.188	6.166	1.328	9.138	1.000
VEpT - VgC	4.166	0.314	13.275	17.764	< .001
VEpT - VgS	-15.643	0.636	-24.579	11.755	< .001
VEpT - VgT	4.089	0.400	10.233	16.401	< .001
VEpT - VpC	3.697	0.599	6.176	12.153	.011

Comparison	Mean Difference	SE	t	df	p_{tukey}
VEpT - VpS	-17.407	7.240	-2.404	9.019	.872
VEpT - VpT	3.946	0.275	14.327	14.995	< .001
VEpT - VEpTS	-3.805	0.439	-8.675	15.201	< .001
VEpT - TpTS	-3.692	0.278	-13.280	15.288	< .001
VEpT - BpTS	-1.416	0.406	-3.485	16.188	.359
VEpT - CpTS	-3.555	0.684	-5.196	11.350	.046
VEpT - VpTS	-3.760	2.062	-1.823	9.235	.988
VEpT - SpTS	-2.223	1.175	-1.891	9.744	.984
VEpT - VEgTS	-3.914	0.387	-10.111	16.802	< .001
VEpT - TgTS	-4.514	0.301	-14.974	17.250	< .001
VEpT - BgTS	-1.459	0.382	-3.815	16.949	.225
VEpT - CgTS	-4.112	0.474	-8.682	14.281	< .001
VEpT - VgTS	-3.043	0.589	-5.166	12.269	.042
VEpT - SgTS	-5.270	0.587	-8.978	12.295	< .001
VgC - VgS	-19.809	0.627	-31.570	11.205	< .001
VgC - VgT	-0.076	0.385	-0.198	15.380	1.000
VgC - VpC	-0.468	0.589	-0.794	11.531	1.000
VgC - VpS	-21.573	7.239	-2.980	9.015	.627
VgC - VpT	-0.219	0.254	-0.863	16.032	1.000
VgC - VEpTS	-7.970	0.425	-18.733	14.183	< .001
VgC - TpTS	-7.857	0.257	-30.602	16.314	< .001
VgC - BpTS	-5.581	0.392	-14.237	15.156	< .001
VgC - CpTS	-7.721	0.676	-11.423	10.876	< .001
VgC - VpTS	-7.925	2.059	-3.849	9.187	.283
VgC - SpTS	-6.388	1.170	-5.458	9.590	.044

Comparison	Mean Difference	SE	t	df	p_{tukey}
VgC - VEgTS	-8.079	0.372	-21.711	15.823	< .001
VgC - TgTS	-8.679	0.282	-30.780	17.837	< .001
VgC - BgTS	-5.624	0.367	-15.314	15.994	< .001
VgC - CgTS	-8.277	0.461	-17.937	13.342	< .001
VgC - VgTS	-7.208	0.579	-12.443	11.626	< .001
VgC - SgTS	-9.435	0.577	-16.347	11.648	< .001
VgS - VgT	19.732	0.674	29.256	13.944	< .001
VgS - VpC	19.341	0.809	23.922	17.909	< .001
VgS - VpS	-1.764	7.261	-0.243	9.120	1.000
VgS - VpT	19.590	0.609	32.157	10.073	< .001
VgS - VEpTS	11.838	0.698	16.953	15.123	< .001
VgS - TpTS	11.952	0.610	19.582	10.145	< .001
VgS - BpTS	14.228	0.678	20.971	14.151	< .001
VgS - CpTS	12.088	0.874	13.835	17.878	< .001
VgS - VpTS	11.884	2.132	5.573	10.491	.033
VgS - SpTS	13.421	1.295	10.365	13.442	< .001
VgS - VEgTS	11.729	0.667	17.582	13.546	< .001
VgS - TgTS	11.129	0.621	17.911	10.829	< .001
VgS - BgTS	14.185	0.664	21.348	13.396	< .001
VgS - CgTS	11.532	0.721	15.999	16.056	< .001
VgS - VgTS	12.601	0.801	15.724	17.854	< .001
VgS - SgTS	10.374	0.800	12.970	17.840	< .001
VgT - VpC	-0.392	0.639	-0.613	14.552	1.000
VgT - VpS	-21.497	7.244	-2.968	9.036	.633
VgT - VpT	-0.143	0.355	-0.403	12.461	1.000

Comparison	Mean Difference	SE	t	df	p_{tukey}
VgT - VEpTS	-7.894	0.492	-16.038	17.678	< .001
VgT - TpTS	-7.781	0.357	-21.821	12.675	< .001
VgT - BpTS	-5.505	0.464	-11.875	17.989	< .001
VgT - CpTS	-7.644	0.720	-10.621	13.288	< .001
VgT - VpTS	-7.849	2.074	-3.784	9.449	.299
VgT - SpTS	-6.312	1.196	-5.276	10.413	.048
VgT - VEgTS	-8.003	0.447	-17.908	17.956	< .001
VgT - TgTS	-8.603	0.375	-22.932	14.527	< .001
VgT - BgTS	-5.548	0.443	-12.528	17.916	< .001
VgT - CgTS	-8.201	0.524	-15.662	17.053	< .001
VgT - VgTS	-7.132	0.630	-11.322	14.722	< .001
VgT - SgTS	-9.359	0.628	-14.903	14.759	< .001
VpC - VpS	-21.105	7.257	-2.908	9.104	.660
VpC - VpT	0.249	0.570	0.437	10.237	1.000
VpC - VEpTS	-7.502	0.664	-11.297	15.765	< .001
VpC - TpTS	-7.389	0.571	-12.944	10.319	< .001
VpC - BpTS	-5.113	0.643	-7.950	14.771	< .001
VpC - CpTS	-7.252	0.847	-8.566	17.588	< .001
VpC - VpTS	-7.457	2.121	-3.515	10.295	.385
VpC - SpTS	-5.920	1.277	-4.637	12.914	.083
VpC - VEgTS	-7.611	0.631	-12.058	14.128	< .001
VpC - TgTS	-8.211	0.583	-14.093	11.103	< .001
VpC - BgTS	-5.156	0.628	-8.206	13.967	< .001
VpC - CgTS	-7.809	0.688	-11.356	16.659	< .001
VpC - VgTS	-6.740	0.772	-8.734	17.993	< .001

Comparison	Mean Difference	SE	t	df	p_{tukey}
VpC - SgTS	-8.967	0.770	-11.644	17.990	< .001
VpS - VpT	21.354	7.238	2.950	9.007	.641
VpS - VEpTS	13.603	7.246	1.877	9.047	.984
VpS - TpTS	13.716	7.238	1.895	9.008	.982
VpS - BpTS	15.992	7.244	2.208	9.038	.929
VpS - CpTS	13.852	7.265	1.907	9.142	.981
VpS - VpTS	13.648	7.521	1.815	10.433	.991
VpS - SpTS	15.185	7.328	2.072	9.456	.960
VpS - VEgTS	13.494	7.243	1.863	9.033	.985
VpS - TgTS	12.894	7.239	1.781	9.012	.991
VpS - BgTS	15.949	7.243	2.202	9.031	.931
VpS - CgTS	13.296	7.248	1.834	9.058	.987
VpS - VgTS	14.365	7.257	1.980	9.100	.972
VpS - SgTS	12.138	7.256	1.673	9.100	.996
VpT - VEpTS	-7.751	0.398	-19.474	11.681	< .001
VpT - TpTS	-7.638	0.208	-36.698	17.981	< .001
VpT - BpTS	-5.362	0.362	-14.810	12.305	< .001
VpT - CpTS	-7.501	0.659	-11.384	9.910	< .001
VpT - VpTS	-7.706	2.054	-3.752	9.090	.314
VpT - SpTS	-6.169	1.161	-5.315	9.284	.055
VpT - VEgTS	-7.860	0.340	-23.090	12.788	< .001
VpT - TgTS	-8.460	0.239	-35.463	16.830	< .001
VpT - BgTS	-5.405	0.335	-16.130	12.923	< .001
VpT - CgTS	-8.058	0.436	-18.470	11.193	< .001
VpT - VgTS	-6.989	0.559	-12.492	10.285	< .001

Comparison	Mean Difference	SE	t	df	p_{tukey}
VpT - SgTS	-9.216	0.557	-16.538	10.296	< .001
VEpTS - TpTS	0.113	0.400	0.283	11.853	1.000
VEpTS - BpTS	2.389	0.498	4.802	17.783	.042
VEpTS - CpTS	0.250	0.742	0.337	14.392	1.000
VEpTS - VpTS	0.045	2.082	0.022	9.589	1.000
VEpTS - SpTS	1.582	1.210	1.308	10.846	1.000
VEpTS - VEgTS	-0.109	0.482	-0.226	17.416	1.000
VEpTS - TgTS	-0.709	0.416	-1.703	13.407	.997
VEpTS - BgTS	2.346	0.478	4.905	17.297	.037
VEpTS - CgTS	-0.307	0.554	-0.554	17.807	1.000
VEpTS - VgTS	0.762	0.655	1.163	15.936	1.000
VEpTS - SgTS	-1.465	0.653	-2.241	15.974	.946
TpTS - BpTS	2.276	0.364	6.253	12.511	.009
TpTS - CpTS	0.137	0.660	0.207	9.971	1.000
TpTS - VpTS	-0.068	2.054	-0.033	9.096	1.000
TpTS - SpTS	1.469	1.161	1.265	9.303	1.000
TpTS - VEgTS	-0.222	0.342	-0.649	13.018	1.000
TpTS - TgTS	-0.822	0.242	-3.405	17.073	.393
TpTS - BgTS	2.233	0.337	6.622	13.158	.004
TpTS - CgTS	-0.420	0.438	-0.959	11.336	1.000
TpTS - VgTS	0.649	0.561	1.157	10.370	1.000
TpTS - SgTS	-1.578	0.559	-2.825	10.382	.701
BpTS - CpTS	-2.139	0.723	-2.957	13.479	.637
BpTS - VpTS	-2.344	2.075	-1.130	9.472	1.000
BpTS - SpTS	-0.807	1.199	-0.673	10.484	1.000

Comparison	Mean Difference	SE	t	df	p_{tukey}
BpTS - VEgTS	-2.498	0.453	-5.517	17.902	.011
BpTS - TgTS	-3.098	0.382	-8.106	14.312	< .001
BpTS - BgTS	-0.043	0.449	-0.096	17.845	1.000
BpTS - CgTS	-2.696	0.529	-5.099	17.226	.026
BpTS - VgTS	-1.627	0.634	-2.566	14.942	.834
BpTS - SgTS	-3.854	0.632	-6.096	14.980	.007
CpTS - VpTS	-0.205	2.147	-0.095	10.755	1.000
CpTS - SpTS	1.332	1.319	1.010	14.112	1.000
CpTS - VEgTS	-0.359	0.713	-0.503	12.928	1.000
CpTS - TgTS	-0.959	0.670	-1.431	10.555	1.000
CpTS - BgTS	2.096	0.710	2.951	12.793	.640
CpTS - CgTS	-0.556	0.763	-0.729	15.317	1.000
CpTS - VgTS	0.512	0.840	0.610	17.484	1.000
CpTS - SgTS	-1.715	0.838	-2.045	17.459	.981
VpTS - SpTS	1.537	2.350	0.654	14.172	1.000
VpTS - VEgTS	-0.154	2.072	-0.074	9.407	1.000
VpTS - TgTS	-0.754	2.057	-0.367	9.154	1.000
VpTS - BgTS	2.301	2.071	1.111	9.392	1.000
VpTS - CgTS	-0.352	2.090	-0.168	9.725	1.000
VpTS - VgTS	0.717	2.119	0.338	10.247	1.000
VpTS - SgTS	-1.510	2.118	-0.713	10.236	1.000
SpTS - VEgTS	-1.691	1.192	-1.419	10.282	1.000
SpTS - TgTS	-2.291	1.167	-1.963	9.488	.976
SpTS - BgTS	0.764	1.191	0.642	10.234	1.000
SpTS - CgTS	-1.889	1.223	-1.544	11.262	.999

Comparison	Mean Difference	SE	t	df	p_{tukey}
SpTS - VgTS	-0.820	1.272	-0.645	12.780	1.000
SpTS - SgTS	-3.047	1.271	-2.397	12.751	.892
VEgTS - TgTS	-0.600	0.362	-1.658	14.963	.999
VEgTS - BgTS	2.455	0.432	5.689	17.993	.008
VEgTS - CgTS	-0.198	0.514	-0.384	16.677	1.000
VEgTS - VgTS	0.871	0.622	1.400	14.292	1.000
VEgTS - SgTS	-1.356	0.620	-2.186	14.328	.954
TgTS - BgTS	3.055	0.357	8.563	15.136	< .001
TgTS - CgTS	0.402	0.453	0.888	12.657	1.000
TgTS - VgTS	1.471	0.573	2.569	11.183	.820
TgTS - SgTS	-0.756	0.571	-1.324	11.201	1.000
BgTS - CgTS	-2.653	0.511	-5.195	16.520	.024
BgTS - VgTS	-1.584	0.619	-2.558	14.128	.835
BgTS - SgTS	-3.811	0.617	-6.175	14.164	.007
CgTS - VgTS	1.069	0.679	1.573	16.812	1.000
CgTS - SgTS	-1.158	0.677	-1.709	16.845	.998
VgTS - SgTS	-2.227	0.763	-2.920	18.000	.659

Note. Results based on uncorrected means.
